# Advances in different adult stem cell-derived exosomal non-coding RNAs for the treatment of neurological disorders: a narrative review

**DOI:** 10.3389/fcell.2024.1459246

**Published:** 2024-10-09

**Authors:** Lebin Ke, Yingying Cao, Zhiwei Lu, Jamal Hallajzadeh

**Affiliations:** ^1^ Department of Health Examination, The Third Affiliated Hospital of Shanghai University, Wenzhou No. 3 Clinical Institute Affiliated to Wenzhou Medical University, Wenzhou People’s Hospital, Wenzhou, China; ^2^ Department of Neurology, Tiantai People’s Hospital of Zhejiang Province, Tiantai Branch of Zhejiang Provincial People’s Hospital, Hangzhou Medical College, Taizhou, Zhejiang, China; ^3^ Hangzhou Heyunjia Hospital, Hangzhou, Zhejiang, China; ^4^ Department of Biochemistry and Nutrition, Research Center for Evidence-Based Health Management, Maragheh University of Medical Sciences, Maragheh, Iran

**Keywords:** stem cell-derived exosome, ncRNAs, neurological diseases, treatment, exosomes

## Abstract

Neurological disorders are being increasingly recognized as major causes of death and disability around the world. Neurological disorders refer to a broad range of medical conditions that affect the brain and spinal cord. These disorders can have various causes, including genetic factors, infections, trauma, autoimmune reactions, or neurodegenerative processes. Each disorder has its own unique symptoms, progression, and treatment options. Optimal communication between interneurons and neuron-glia cells within the homeostatic microenvironment is of paramount importance. Within this microenvironment, exosomes play a significant role in promoting intercellular communication by transferring a diverse cargo of contents, including proteins, lipids, and non-coding RNAs (ncRNAs). Partially, nervous system homeostasis is preserved by various stem cell-derived exosomal ncRNAs, which include circular RNAs (circRNAs), long noncoding RNAs (lncRNAs), microRNAs (miRNAs), and PIWI-interacting RNAs (piRNAs). The diversity of these exosomal ncRNAs suggests their potential to influence multiple pathways and cellular processes within the nervous system. Stem cell-derived exosomes and their ncRNA contents have been investigated for potential therapeutic uses in neurological disorders, owing to their demonstrated capabilities in neuroprotection, neuroregeneration, and modulation of disease-related pathways. The ability of stem cell-derived exosomes to cross the blood-brain barrier makes them a promising delivery vehicle for therapeutic ncRNAs. This review aims to summarize the current understanding of different stem cell-derived exosomal ncRNAs and their therapeutic potential and clinical applications.

## 1 Introduction

Neurological disorders impact hundreds of millions of people around the world (Huang Y. et al., 2023). Neurological disorders encompass a wide range of conditions that impact the central, peripheral, and autonomic nervous systems of the body. Neurological disorders are a leading cause of disability and death worldwide (Huang Y. et al., 2023). Conditions like Alzheimer’s, Parkinson’s, stroke, and traumatic brain injuries can significantly impair quality of life and lead to premature mortality. The global prevalence of neurological disorders is projected to increase in the coming decades (Huang Y. et al., 2023). This is driven by aging populations, especially in low- and middle-income countries where the burden is often highest. The economic costs associated with neurological diseases are staggering, estimated at over $800 billion per year globally (Huang Y. et al., 2023). This includes direct medical costs as well as indirect costs from lost productivity. The challenges facing patients with nervous system disorders include irreversible damage, cognitive impairment, and resistance to treatment (Huang Y. et al., 2023). Additionally, the limited knowledge of the molecular causes of these conditions and the lack of early diagnostic tests and sensitive tools to monitor treatment effectiveness have significantly impeded available interventions, resulting in very poor prognoses for those with nervous system diseases. Improving our understanding of the molecular processes driving the development and progression of nervous system diseases, as well as promoting research into early diagnostic methods and new treatment approaches, are absolutely critical to addressing the substantial burden posed by these devastating neurological disorders.

Despite extensive research efforts, the limited engraftment and survival of transplanted stem cells continue to be significant challenges that must be overcome in order to improve the clinical efficacy of stem cell-based treatments for neurological disorders (Hoang et al., 2022; Temple, 2023; Zhang and Cheng, 2023). Recent research has shown that stem cells primarily exert their therapeutic effects on neurological diseases through the release of extracellular vesicles known as exosomes (Zhang and Cheng, 2023; Fayazi et al., 2021). These exosomes are rich in bioactive molecules, such as proteins, lipids, and ncRNAs, which collectively contribute to intercellular signaling and modulate various physiological processes. By enhancing cell survival, reducing inflammation, and promoting tissue repair, exosomes play a critical role in the regenerative potential of stem cells, offering promising avenues for the treatment of neurological disorders (Tan et al., 2024). Although the proportion of ncRNAs in Exo is relatively low, research shows that these ncRNAs, especially those present in stem cell-derived Exo, contribute significantly to the treatment of nervous system diseases by neuroprotection (Zhang L. et al., 2024),anti-apoptosis (Cheng et al., 2024), neuroplasticity and regeneration (Zhong et al., 2023), immunomodulation and anti-inflammation (Shan et al., 2023), epigenetic regulation and intercellular communication (Dragomir et al., 2018). For example, exosomal miR-146a-5p from umbilical cord-derived MSCs can suppress the activation of microglia and astrocytes, reducing neuroinflammation in models of ischemic stroke (Zhang Z. et al., 2021). Exosomal lncRNA MALAT1 from adipose-derived stem cells can stimulate the differentiation of neural stem/progenitor cells and enhance neurogenesis in the injured brain (El Bassit et al., 2017).

Adult-derived stem cells are more readily available, simpler to acquire, and do not present the same ethical dilemmas as stem cells derived from embryonic sources (Zakrzewski et al., 2019). Developing therapeutic approaches that utilize adult stem cell-derived exosomes and their non-coding RNAs is of utmost importance, as this holds great promise for treating a diverse range of debilitating neurological diseases, which pose a significant global health challenge. This review systematically explores the research progress and the underlying mechanisms by which diverse adult stem cell-derived exosomal ncRNAs can be leveraged for the treatment of nervous system diseases.

## 2 Stem cells

**FIGURE 1:**
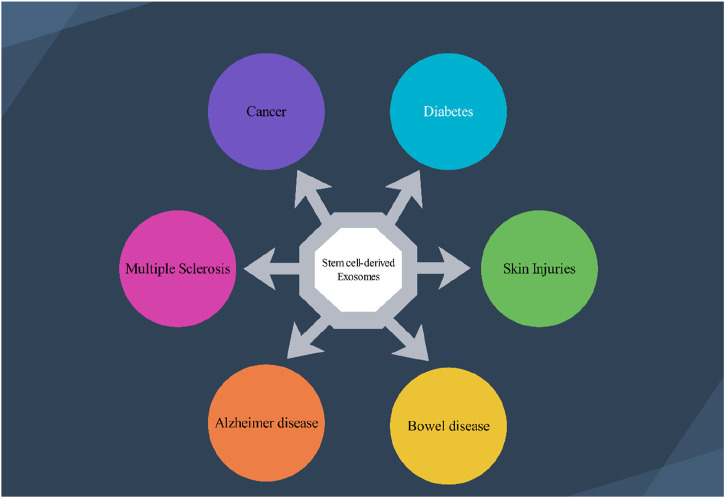
Several stem cell–derived exosomes therapeutic application in various disease. 1.1) Exosomes deliver neuroprotective factors, facilitating neuronal survival and regeneration in conditions like Alzheimer’s and Multiple Sclerosis’s disease. 1.2) Stem cell-derived exosomes can modulate the tumor microenvironment, potentially inhibiting tumor growth and enhancing the efficacy of chemotherapeutics. 1.3) Stem cell-derived exosomes are shown to improve insulin sensitivity and promote pancreatic beta-cell regeneration. 1.4) Stem cell-derived exosomes facilitate wound healing by promoting skin cell proliferation, angiogenesis, and reducing inflammation, thus enhancing the repair of damaged skin. 1.5) Exosomes play a role in modulating inflammation and promoting mucosal healing in conditions such as inflammatory bowel disease (IBD), aiding in the restoration of gut integrity.

Stem cells are a group of unspecialized cells that have the remarkable capacity to continually divide and renew themselves, as well as the potential to develop into various functional cell types that make up different tissues and organs (Zakrzewski et al., 2019). Stem cells are primarily categorized based on their ability to differentiate into different cell types, the source or origin from which they are derived, and their developmental progression or lineage (Zakrzewski et al., 2019). Stem cells can be classified according to their degree of potency, or ability to differentiate into different cell types (Zakrzewski et al., 2019). They range from totipotent cells that can give rise to all cell types, to pluripotent cells that can form many but not all cell types, to multipotent, oligopotent and unipotent cells that can form more limited sets of specialized cell type (Zakrzewski et al., 2019). Stem cells are found in both embryonic and adult tissues. The most prominent examples of pluripotent stem cells are embryonic stem cells (ESCs) (Calabrese, 2022) and induced pluripotent stem cells (iPSCs) (Aboul-Soud et al., 2021). On the other hand, adult tissues contain multipotent stem cells such as hematopoietic stem cells (HSCs) (Carroll and St Clair, 2018), mesenchymal stem cells (MSCs) (Samsonraj et al., 2017), neural stem cells (NSCs) (Urbán et al., 2019), and endothelial stem/progenitor cells (EPCs) (Chong et al., 2016). The various subtypes of stem cells, including embryonic, induced pluripotent, and adult multipotent stem cells, have been extensively studied and trialed as potential treatments for a wide range of human diseases. Exosomes are considered miniature versions or representations of their parent cells, in part because exosomes from a particular cell type contain cell-specific or unique sets of biomolecules. Additionally, stem cells have been found to function in a paracrine manner, meaning they act through the soluble factors they secrete, including exosomes, rather than solely through direct cell-cell interactions. In essence, the exosomes secreted by stem cells (SC-Exo) inherit similar therapeutic benefits as their parental stem cells, such as anti-inflammatory effects, immunomodulatory properties, and the ability to promote tissue regeneration (Tan et al., 2024). Stem cells have distinct ncRNA expression profiles that influence their fate and differentiation pathways (Marson et al., 2008). These unique expression profiles can be harnessed to guide the terminal differentiation of somatic cells from stem cells, providing a powerful strategy for regenerative medicine (Yu et al., 2023). By leveraging specific ncRNAs associated with particular cell types, researchers can effectively direct stem cells to develop into specialized cells. This approach holds significant promise for treating a variety of diseases, including muscle diseases, cardiovascular diseases, and neurological diseases. Stem cell-derived exosomes can share some common ncRNA profiles, but they also exhibit significant variability depending on the type of stem cell and the specific conditions under which they are derived (Tan et al., 2024). Many types of stem cells (e.g., embryonic stem cells, mesenchymal stem cells) may express certain core ncRNAs, such as specific miRNAs that are involved in regulating stemness, proliferation, and differentiation (Tan et al., 2024). The differences in ncRNAs among exosomes from various stem cell types can lead to distinct biological effects. For example, exosomes from mesenchymal stem cells might promote tissue repair, while those from neural stem cells might enhance neuroprotection (Tan et al., 2024). The local microenvironment and the specific stimuli (e.g., hypoxia, inflammation) can influence the composition of ncRNAs in exosomes, leading to differential effects on recipient cells. Each stem cell type has its unique transcriptional and post-transcriptional regulation mechanisms, leading to different ncRNA profiles. The presence of specific growth factors, cytokines, and other signaling molecules can alter ncRNA expression in stem cells, thus affecting the composition of their derived exosomes. The differentiation state of the stem cells (e.g., undifferentiated vs. differentiated) can also influence the ncRNA profiles, as the cells adapt their exosomal content to their functional roles. Epigenetic factors can play a significant role in regulating the expression of ncRNAs, contributing to the variability seen in exosome content among different stem cell types. However, using ncRNAs from exosomes derived from different stem cells has advantages when used as agents for neurological disorders (Pishavar et al., 2022). One advantage of exosomes derived from stem cells is that they can effectively pass through the BBB, enabling them to transmit ncRNAs to the nervous system. Moreover, by encapsulating the ncRNAs within exosomes, they become more stable and protected from degradation. Furthermore, exosomes derived from stem cells have features that can help in the regeneration of tissues, offer protection to the brain, and influence the microenvironment affected by diseases in the brain (Pishavar et al., 2022). This review focuses on understanding the role of ncRNAs obtained from exosomes derived from different stem cells in the treatment of various neurological disorders. The article emphasizes the importance of conducting research and investigation into these ncRNAs. Such exploration holds the potential for developing treatment strategies focusing on improving the healing process and minimizing complications in individuals with neurological disorders. Researchers are currently exploring the applications of stem cell-derived exosomes in neurological diseases, skin injuries, gastrointestinal diseases, and cancer (Figure 1). To better understand the functionality of these stem cell-derived exosomal ncRNAs-based therapies, further research is needed to develop improved techniques for the production and purification of the ncRNAs, as well as to establish robust protocols for their therapeutic delivery.

## 3 Exosomes biogenesis

**FIGURE 2:**
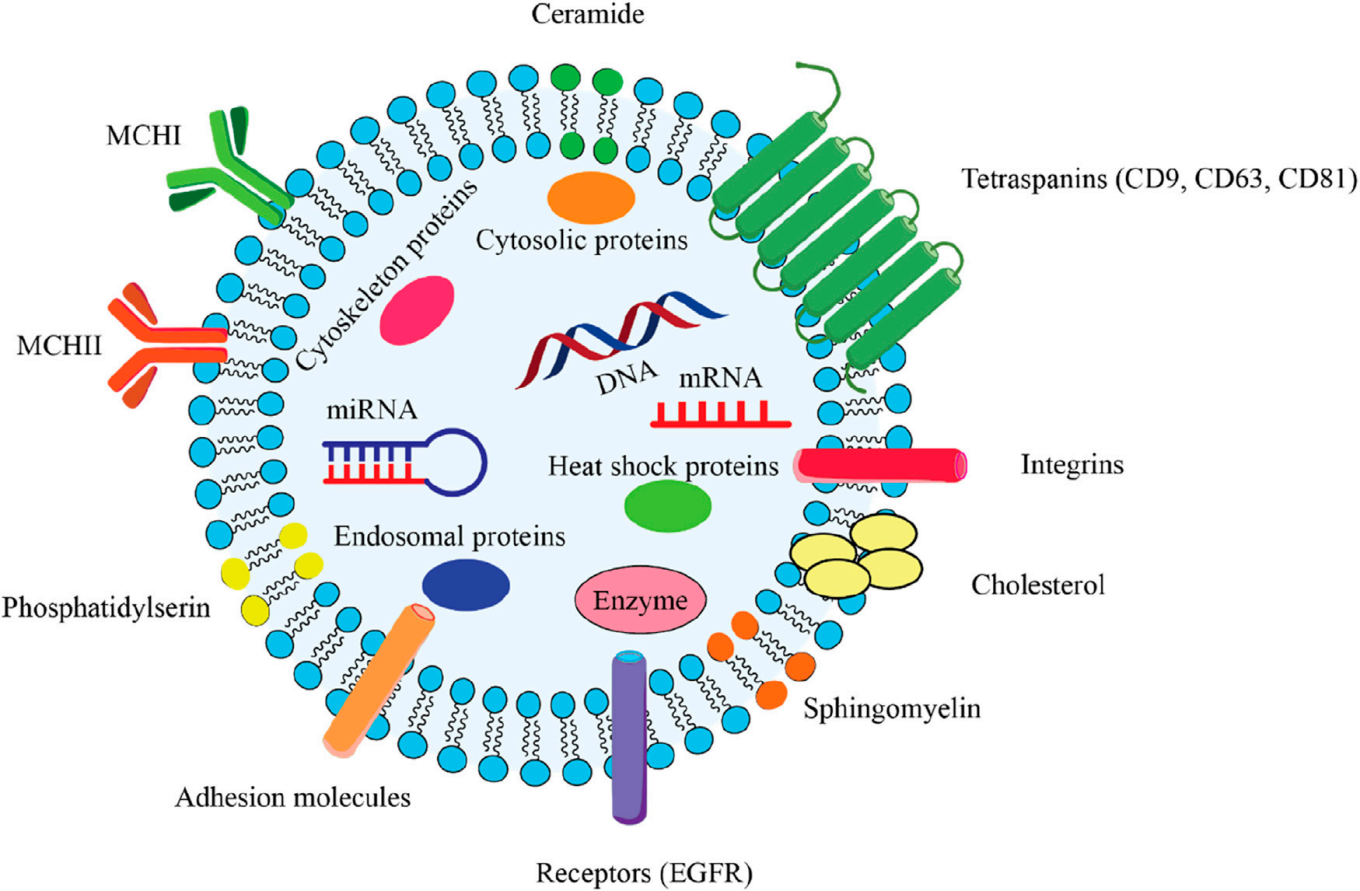
Comprehensive overview of the key structural components of an exosome, which is a type of extracellular vesicle involved in intercellular communication. 2.1) Lipid bilayer membrane: The outer structure of the exosome, composed of a phospholipid bilayer that encases the internal cargo and maintains the integrity of the vesicle. 2.2) Encapsulated Cargo: mRNA and miRNA play critical roles in gene regulation and protein synthesis. DNA that can be transferred between cells. Cytosolic proteins include various proteins that are important for cellular function and signaling. Endosomal proteins derived from the endosomal pathway, essential for exosome biogenesis. Enzymes catalysts biochemical reactions that may influence recipient cells. 2.3) Surface Markers: Tetraspanins (CD9, CD63, CD81) facilitate exosome formation and play a role in cell recognition and adhesion. Integrins mediate interactions with the extracellular matrix and other cells. Adhesion molecules that assist in the binding of exosomes to target cells. Receptor (EGFR) can mediate cellular responses upon binding with ligands. 2.4) Lipid Components: Ceramide is a sphingolipid that contributes to membrane stability and signaling. Cholesterol is a critical component for maintaining membrane fluidity and structure. Phosphatidylserine is a phospholipid that is involved in cell signaling and recognition. Sphingomyelin is a type of sphingolipid that plays a role in membrane structure and function. 2.5) Heat Shock Proteins: These proteins are involved in protecting cells from stress and facilitating protein folding.

**FIGURE 3:**
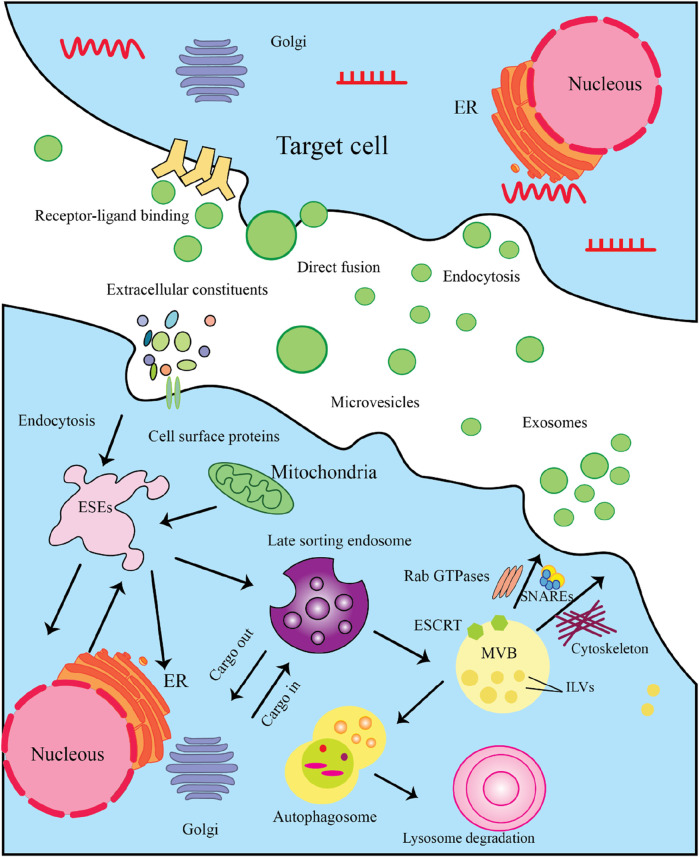
The Multistep Process of Exosome Biogenesis and Secretion. Here’s a detailed explanation of the steps involved. 3.1) Cargo Synthesis and Sorting: The process begins in the nucleus, where mRNAs and proteins are synthesized. These molecules are then translated in the endoplasmic reticulum (ER). After synthesis, proteins are transported to the Golgi complex for post-translational modifications and sorting. 3.2) Formation of Exosome-Secretory Endosomes (ESEs): Cells can internalize extracellular constituents through endocytosis. This process allows the uptake of cellular materials, including proteins and lipids, which may be incorporated into exosomes. Some membrane protrusions can shed from the cell surface, forming microvesicles that may also participate in intercellular communication. 3.3) Late Sorting Endosome: Internalized materials are directed to late endosomes, which serve as sorting hubs for cargo destined for exosomes. Here, the endosomal membrane invaginates to form intraluminal vesicles (ILVs). The Endosomal Sorting Complex Required for Transport (ESCRT) is crucial for the budding of ILVs within late endosomes, facilitating the encapsulation of proteins and nucleic acids into these vesicles. 3.4) MVB Formation: As ILVs accumulate within late endosomes, they transform into multivesicular bodies (MVBs). MVBs can either fuse with lysosomes for degradation or with the plasma membrane for exosome release. 3.5) Exosome Secretion: MVBs that are destined to release exosomes will fuse with the plasma membrane, allowing ILVs to be expelled as exosomes into the extracellular space. In some cases, exosomes may also enter target cells by directly fusing with the cell membrane, releasing their cargo. 3.5) Target Cell Interaction: Once in the extracellular environment, exosomes can interact with target cells through specific receptor-ligand binding, which initiates signaling pathways. Target cells can uptake exosomes through endocytosis, allowing the internalization of their bioactive molecules.

Exosomes originating from stem cells have shown promise in the treatment of various conditions (Zhang Q. et al., 2024; Huang Y. et al., 2023; Hoang et al., 2022). Exosomes are a specific subset of the broader category of extracellular vesicles (EV), distinguished by their smaller size range of 30–100 nm in diameter (Temple, 2023). The EV family encompasses several different types of membrane-bound structures, each with their own unique properties and functions. Exosomes carry a diverse cargo of biomolecules, including proteins, lipids, and nucleic acids, that are reflective of the parent cell that produced the exosome. This biomolecular content within exosomes can function as distinctive “markers” or “signatures” that provide information about the cell of origin. Exosomes commonly contain protein markers such as tetraspanins (CD9, CD63, CD81) and membrane transport/fusion proteins like Rab GTPases and SNAREs (Figure 2) (Temple, 2023). Heat shock proteins are also frequently detected as exosomal markers (Temple, 2023). The specific molecular composition of exosomes reflects the unique makeup of the parent cell, providing valuable information about the state and origin of the exosomes. Exosomes are formed and secreted through a multi-step process. Recent evidence indicates that exosomes are generated not only through the endosomal pathway, but also via a process of membrane invagination. The exosome formation process begins with the internalization of various extracellular materials, including proteins, lipids, metabolites, and cell membrane components, into the cell via endocytosis (Figure 3) (Zhang and Cheng, 2023). The internalization occurs through the invagination, or inward folding, of the cell membrane, creating primary endocytic vesicles that enclose the extracellular materials. The primary endocytic vesicles then fuse together to form early endosomes (EEs) within the cell. These early endosomes subsequently undergo a maturation process, transforming and developing into late endosomes (LEs). As the late endosomes mature, their internal membrane begins forming multiple invaginations or inward folds within the lumen of the organelle. The selective membrane invaginations within the late endosomes encapsulate and sort specific proteins, lipids, nucleic acids, and other cellular components, packaging them into intraluminal vesicles (ILVs) inside the organelle. The late endosomes containing the ILVs then further develop and mature into multivesicular bodies (MVBs). Specific MVBs are directed towards the cell’s outer membrane by intracellular transport mechanisms. These MVBs then merge with the plasma membrane, releasing their internal vesicle contents into the extracellular space. These released vesicles are now classified as exosomes, which can be absorbed by nearby cells or continue to circulate outside the cell. Some MVBs are transported to and merge with the cell’s lysosomes. This enables the contents of the MVBs, including any unwanted or damaged materials, to be broken down and cleared from the cell. The process is regulated by a coordinated effort between key proteins, including RAB GTPases, tethering factors, SNARE proteins, and cytoskeletal proteins. These proteins work together to ensure the MVBs are properly transported, docked, and fused with the cell’s outer membrane, facilitating the release of exosomes (Zhang and Cheng, 2023). In addition to the regulatory proteins, important environmental factors within the cell - including calcium levels, pH, and oxygen concentrations - also significantly impact the production and release of MVBs and exosomes. Conditions such as high calcium, low pH, and low oxygen levels can all enhance and regulate the formation and release of these extracellular vesicles (Fayazi et al., 2021). The interplay between the specialized proteins involved and the cellular environmental factors is critical for ensuring the proper and directed secretion of exosomes.

## 4 Exosome-derived noncoding RNAs

Exosomes contain a wide range of ncRNA types, including miRNAs, lncRNAs, circRNAs, piRNAs, and siRNAs. As the discovery and study of non-coding RNAs (ncRNAs) in exosomes has advanced, many innovative functions and applications have emerged. These range from novel mechanisms of cell-to-cell communication to the use of exosomal ncRNAs as promising disease biomarkers. Furthermore, exosomal ncRNAs have shown new therapeutic applications, particularly in the context of neurological diseases. The incorporation of specific ncRNAs into exosomes is a highly regulated process, involving various cellular mechanisms and pathways. For instance, the endosomal sorting complex required for transport (ESCRT) proteins play a crucial role in the incorporation of miRNAs into intraluminal vesicles (ILVs), which are then released as exosomes (Henne et al., 2011; Frankel and Audhya, 2018). The ESCRT machinery is involved in the budding and scission of the ILVs, providing a mechanism for the selective packaging of miRNAs into these vesicles (Frankel and Audhya, 2018). Also, the neutral sphingomyelinase 2 (nSMase2) enzyme has been implicated in the sorting of miRNAs into exosomes (Frankel and Audhya, 2018). nSMase2 catalyzes the hydrolysis of sphingomyelin to ceramide, which can promote the formation of lipid-enriched microdomains in the endosomal membrane. These ceramide-enriched microdomains may then facilitate the recruitment and packaging of miRNAs into the ILVs. These two pathways highlight the complex and regulated nature of the processes involved in the loading of specific ncRNA species into these extracellular vesicles. The detailed mechanisms underlying the selective packaging of various ncRNA species into exosomes require further exploration and research.

### 4.1 microRNAs

miRNAs are short, ncRNA molecules that do not encode proteins, typically ranging from 19 to 25 nucleotides in length, and play regulatory roles in eukaryotic organisms (Ranganathan and Sivasankar, 2014). In the nucleus, the genes encoding miRNAs are transcribed into long initial transcripts (pri-miRNAs), which are then processed by the Drosha and DGCR8 proteins to produce a stem-loop structure called pre-miRNA, approximately 70 nucleotides long (Ranganathan and Sivasankar, 2014; Michlewski and Cáceres, 2019). This pre-miRNA is further cleaved by the Dicer enzyme in the cytoplasm to generate the mature miRNA. The mature miRNA is then exported from the nucleus and, with the help of a helicase, can assemble into the RNA-induced silencing complex (RISC) (Iwakawa and Tomari, 2022). Within the RISC complex, the miRNA can regulate gene expression by base pairing with complementary sequences on target mRNAs, leading to mRNA degradation or translational repression.

### 4.2 Long non-coding RNAs

lncRNAs are a class of ncRNAs that are longer than 200 nucleotides, with some exceeding thousands of nucleotides in length (Han and Chen, 2024). Unlike miRNAs, lncRNAs can adopt complex secondary and tertiary structures, which are important for their diverse functional roles. The subcellular localization of lncRNAs is an important factor that determines their diverse functional roles in eukaryotic cells. In the cell nucleus, lncRNAs can participate in diverse regulatory functions, including transcriptional regulation, organization of chromatin structure, and regulation of RNA splicing (Han and Chen, 2024). In the cytoplasm, such as in exosomes, they can serve different roles, including post-transcriptional regulation, sequestration of microRNAs, and various signaling functions (Han and Chen, 2024). Concurrently, the expanding knowledge about exosomal long non-coding RNAs has driven increased focus on understanding their roles in biology. This has steadily revealed that exosomal lncRNAs play a significant part in the treatment of many nervous system diseases.

### 4.3 CircRNAs

CircRNAs are a form of non-coding RNA that are produced through a reverse splicing mechanism and reside within the cell nucleus (Feng et al., 2023). Certain circRNAs are derived from the non-coding segments of genes, while others include at least one protein-coding region and are predominantly located in the cell’s cytoplasm (Feng et al., 2023). CircRNAs are abundantly present in tissues and cell types, including the brain and have been conserved across different species throughout evolution. Recent research has revealed that exosomal circular RNAs can serve multiple functions, including acting as regulating splicing and transcription, inhibiting proteins, and miRNA sponges. They play roles in biological processes and are implicated in several disease conditions. In the field of neurobiology, specifically circRNAs, there is an increasing body of reports suggesting their role in the progression of disorders such as Parkinson’s disease, schizophrenia, Alzheimer’s disease, and epilepsy. Researchers suggests that circRNAs are beneficial in identifying and addressing these conditions as both biomarkers (Cheng et al., 2024) and treatment targets (Cheng et al., 2024; El Bassit et al., 2017).

### 4.4 piRNAs

piRNAs are a class of small non-coding RNA molecules that interact with PIWI proteins (Cheng et al., 2024). piRNAs guide PIWI proteins to complementary sequences, leading to the degradation or transcriptional silencing of target RNAs (Wu et al., 2023). The biogenesis of piRNAs involves a distinct pathway from miRNA or siRNA production and are thought to be important for germ cell development, stem cell maintenance, and epigenetic regulation (Wu et al., 2023). In contrast to microRNAs, piRNAs have the ability to silence genes. They can participate in diverse processes, such as transcriptional silencing or activation, transposon silencing, post-transcriptional regulation, and other modifications (Wu et al., 2023). This highlights the unique characteristics of piRNAs compared to other types of non-coding RNAs. piRNAs appear to be important players in maintaining neuronal homeostasis, supporting brain development, and potentially contributing to cognitive processes and neurological health (Zhong et al., 2023).

## 5 NcRNAs from stem cell–derived exosomes and their potential mechanisms in neurological disorders

**FIGURE 4:**
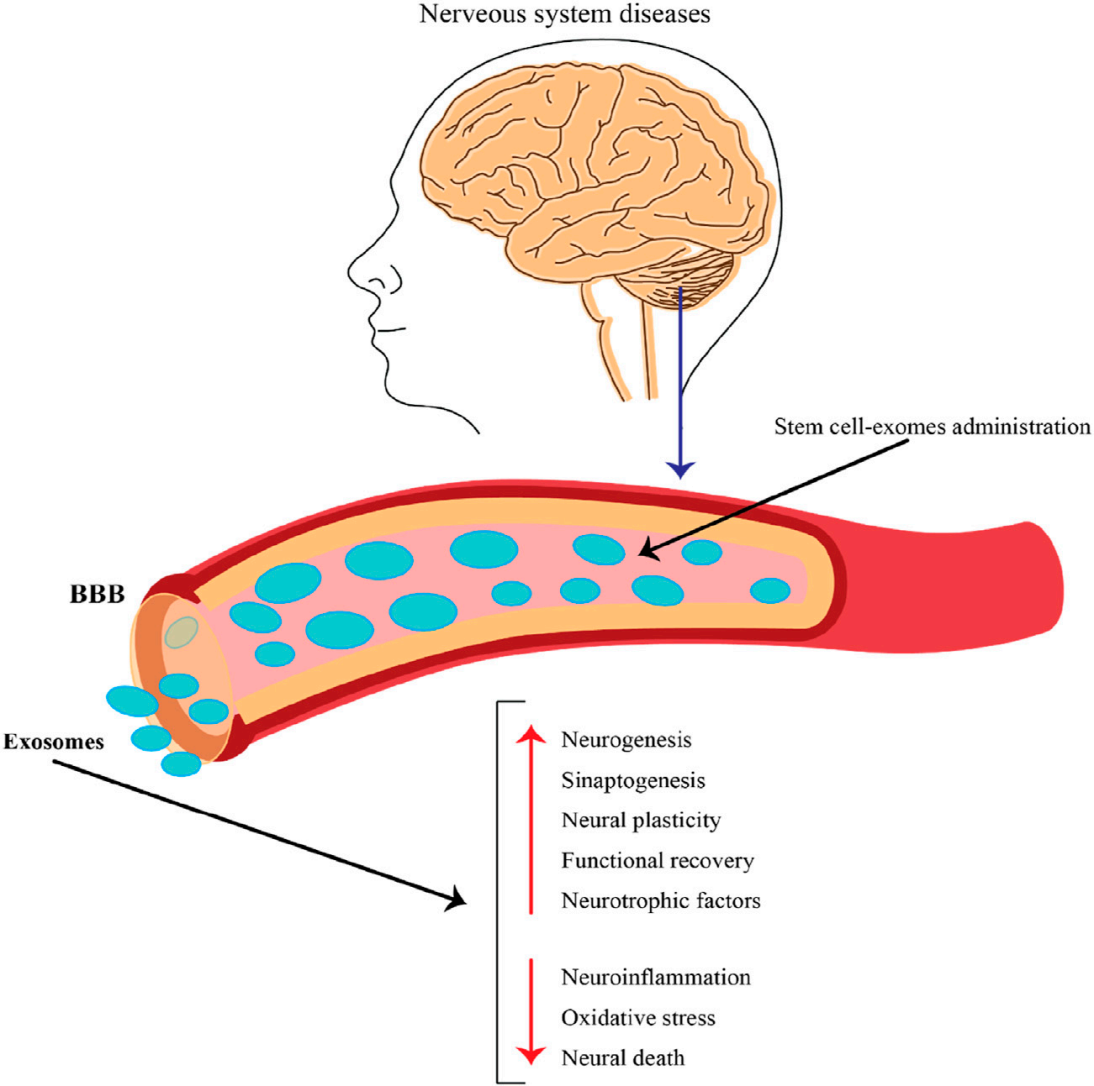
The role of exosomes released by stem cells in the context of nervous system diseases and their potential therapeutic applications. This figure emphasizes the potential of stem cell-derived exosomes as a therapeutic strategy for treating nervous system diseases. By crossing the BBB and delivering beneficial cargo, these exosomes can promote neural repair, enhance recovery, and combat harmful processes in the brain, making them a promising avenue for future research and clinical applications.

NcRNAs obtained from stem cell-derived exosomes can exert therapeutic effects against neurological disorders via different mechanisms. Here are some possible mechanisms by which these ncRNAs from stem cell–derived exosome regulate biological functions and promote neurological outcomes (Figure 4).

### 5.1 Modulation gene expression patterns associated with key neural processes

Stem cell-derived exosome ncRNAs can modulate the expression of specific genes by binding to mRNA molecules (Tian et al., 2022; Bai et al., 2022; Xie et al., 2023) and altering their stability or translation. Through molecular interactions, ncRNAs can influence the expression of genes associated with processes like synaptic plasticity (Pan et al., 2020), neuroprotection (Zhuang et al., 2023; Wang et al., 2021), neuroinflammation (Zhang Z. et al., 2021; Zhang Y. et al., 2021), neurogenesis (Zhang Y. et al., 2021; Pan et al., 2021), and other significant biological processes.

#### 5.1.1 Neuroinflammation

Neurological disorders often exhibit a characteristic called neuroinflammation. This phenomenon occurs when immune cells become active and liberate substances that prompt inflammation. Stem cell-derived exosomes can transport ncRNAs, including miRNAs and lncRNAs, which can specifically target genes associated with immune responses and inflammation (Zhang Z. et al., 2021; Zhang Y. et al., 2021). By controlling the expression of these genes, stem cell-derived exosomal ncRNAs can effectively modify the response in the nervous system and reduce neuroinflammation associated with neurological disorders (Zhang Z. et al., 2021; Zhang Y. et al., 2021).

#### 5.1.2 Neuroprotection

Experimental studies confirmed that exosomes derived from stem cells, along with their ncRNAs content, can support the nervous system in various neurological disorders. These exosomes can transport ncRNAs, which target genes involved in cell survival (El Bassit et al., 2017; Pan et al., 2020; Liu J. et al., 2022), apoptosis, and response to oxidative stress. By modifying the expression of these genes, the ncRNAs carried by exosomes can protect neurons’survival, decrease cell death rates, and increase the resilience of neurons against insults. This effects finally helps to safeguard the system during disease conditions (Chang et al., 2021; Luo et al., 2022).

#### 5.1.3 Synaptic plasticity

Learning, memory, and general brain function are all dependent on synaptic plasticity, or the synapses’ ability to change in strength and connectivity. Exosomes derived from stem cells can convey ncRNAs that regulate the expression of genes implicated in synaptic plasticity (Pan et al., 2020). These genes encode neurotransmitter receptors, synaptic proteins, and signaling molecules. Exosomal ncRNAs may improve synaptic plasticity (Pan et al., 2020) and neuronal connectivity in neurological disorders with synaptic dysfunction by regulating the expression of these genes.

#### 5.1.4 Neurogenesis and differentiation

Neurogenesis is crucial for brain development, learning, and memory formation. Stem cells, which have the ability to differentiate into various cell types, play a crucial role in neurogenesis. NcRNAs containing exosomes derived from stem cells can control the expression of genes that play a role in these processes, encouraging the growth of neurons. The development of spines, the formation of synapses, and the modification of existing synaptic connections. By enhancing these processes, stem cell-derived exosomal ncRNAs can contribute to neuronal regeneration (Zhang L. et al., 2024; Tan et al., 2024; Pishavar et al., 2022; Henne et al., 2011), functional recovery (Zhang L. et al., 2024; Sun and Xu, 2023), and cognitive improvement (Zhang L. et al., 2024; Tan et al., 2024; Huang et al., 2021) in neurological disorders. Stem cell-derived exosomes can transport ncRNAs that target genes involved in NSCs fate determination (Zhang L. et al., 2024; Tan et al., 2024). Adult mammalian neural stem cells possess distinctive characteristics that set them apart from other stem cell types. These key properties include their ability to differentiate into various neural cell lineages, their capacity for self-renewal to maintain the stem cell pool, and their ability to enter a dormant state (Dong et al., 2022). NcRNAs containing exosomes derived from stem cells can stimulate neurogenesis through several key mechanisms. Exosomal ncRNAs can target and modulate the expression of key transcription factors involved in neural lineage specification and neurogenesis, such as SOX2, NEUROG2, ASCL1, and OLIG2. For example, exosomal miR-124 from NSCs can target the 3′ UTR of the SCP1 gene, which in turn upregulates the expression of the neurogenic transcription factor NEUROG2, promoting neuronal differentiation (Zhang et al., 2023). Exosomal ncRNAs can regulate signaling pathways that are crucial for NSCs fate determination, proliferation, and neuronal differentiation, such as the Notch, Wnt, and Sonic Hedgehog pathways. Exosomal ncRNAs can influence epigenetic modifications, such as DNA methylation and histone acetylation, which can alter the expression of genes involved in neurogenesis. Exosomal lncRNA MALAT1 from NSCs has been reported to interact with the polycomb repressive complex 2 (PRC2), leading to the epigenetic silencing of genes that inhibit neuronal differentiation (Zhao C. et al., 2022). Exosomal ncRNAs can target genes and pathways involved in neuronal maturation, dendritic arborization, and synaptic formation and function. Exosomal miR-132 from MSCs has been shown to enhance dendritic spine formation and synaptic plasticity, contributing to the functional integration of newborn neurons (Ma et al., 2022). Specific exosomal ncRNAs can modulate the inflammatory response in the central nervous system, which can influence the neurogenic niche and support the survival and integration of newborn neurons. Exosomal lncRNA NEAT1 from NSCs has been implicated in the regulation of neuroinflammation, potentially promoting a more favorable environment for neurogenesis (Zhang Q. et al., 2024). Exosomal ncRNAs can be transferred to other cell types in the neurogenic niche, such as astrocytes, oligodendrocytes, and endothelial cells, influencing their functions and indirectly supporting neurogenesis. For instance, exosomal miR-21 from NSCs can be taken up by astrocytes, leading to the upregulation of neurotrophic factors that support neuronal survival and differentiation (Zhong et al., 2023).

#### 5.1.5 Blood-brain barrier integrity

BBB plays a critical role in modulating the passage of molecules and cells into the brain. Compromised BBB leads to the development of neurological disorders. However, there is a potential solution using exosomes derived from stem cells. These exosomes convey special ncRNAs that can impact the expression of genes responsible for preserving BBB integrity and function. By modulating these gene expressions, exosomal ncRNAs can effectively support the BBB barrier against substances entering the brain (Wang et al., 2022; Hill, 2019).

### 5.2 Modulation of signaling pathways

Stem cell-derived exosomal ncRNAs can influence critical signaling pathways involved in the development of neurological disorders. As an example, miRNAs have the ability to change the function of signaling pathways, including the Wnt/β-catenin (Xu et al., 2019), AKT/ERK pathways (Zhuang et al., 2023; Jiang et al., 2021), and PI3K/Akt pathways (Pan et al., 2020; Wei et al., 2020). These pathways closely play critical roles in cellular processes such as synaptic plasticity, neurogenesis, and cell survival. By modulating these pathways, ncRNAs can affect cellular processes related to neurological disorders and facilitate neuroprotection and functional recovery. The modification of signaling pathways is a crucial component of cellular function and is frequently disrupted in neurological disorders. Stem cell-derived exosome ncRNAs have been identified to regulate signaling pathways in neurological disorders. The following sections provide an overview of how ncRNAs from different stem cell-derived exosomes affect signaling pathways and their implications for neurological disorders:

#### 5.2.1 Wnt/β-catenin pathway

Disruption in the Wnt/β pathway may affect on brain development, synaptic plasticity, and the generation of neurons. Stem cell-derived exosomes contain ncRNAs that specifically target various elements of the Wnt/β-catenin pathway, such as β-catenin and Wnt ligands. These exosomal ncRNAs can regulate the expression or activity of these targets, consequently influencing the signaling of the Wnt/β-catenin pathway. Consequently, this modulation has implications for safeguarding the health of the human brain, the adapting our connections, and generating new brain cells (Xu et al., 2019).

#### 5.2.2 PI3K/Akt/mTOR pathway

The PI3K/Akt/mTOR pathway plays a critical role in promoting the survival of neurons, their growth, and the plasticity of synapses. Stem cell-derived exosomes can carry ncRNAs that target genes in this pathway, such as PI3K, Akt, and mTOR. By modulating the expression or activity of these genes, exosomal ncRNAs can modulate PI3K/Akt/mTOR signaling, influencing survival, dendritic growth, and synaptic connectivity in neurological disorders (Wei et al., 2020).

#### 5.2.3 AKT/ERK pathways

The AKT/ERK pathway is essential for neuronal development, synaptic plasticity, and neuronal survival. Stem cell-derived exosomes can transport ncRNAs that target components of this pathway, including kinases and transcription factors (Zhuang et al., 2023; Jiang et al., 2021). Stem cell-derived exosomal ncRNAs can influence the expression or function of these elements, resulting in the modulation of AKT/ERK pathway signaling and its subsequent impact on function, synaptic plasticity, and neuroprotection (Zhuang et al., 2023; Wang et al., 2021).

#### 5.2.4 NF-κB pathway

Stem cell-derived exosomal ncRNAs can influence the expression or function of these elements, resulting in the modulation of NF-κB pathways signaling (Zhang Z. et al., 2021; Zhuang et al., 2023; Wang et al., 2021; Jiang and Zhang, 2021; Sun and Xu, 2023) and its subsequent impact on function, synaptic plasticity, and neuroprotection. Exosomes derived from stem cells can transport ncRNAs that specifically target genes involved in the NF-κB pathway, including NF-κB subunits,and upstream regulators (Wang et al., 2021; Jiang and Zhang, 2021). By modulating the expression or activity of these genes, stem cell-derived exosomal ncRNAs can influence NF-κB signaling and modulate neuroinflammation,and immune responses in neurological disorders (Jiang and Zhang, 2021; Sun and Xu, 2023; Fan et al., 2021).

The delivery of ncRNAs containing exosomes derived from stem cells to recipient cells provides a means to regulate the functioning of signaling pathways implicated in neurological disorders. By targeting the critical components of these pathways, ncRNAs containing exosomes derived from stem cells can influence various cellular processes, including neurodevelopment, neuroinflammation, synaptic plasticity (Pan et al., 2020), and neuroprotection. Additional investigations are required to reveal the ncRNAs implicated, their functions, and their potential for therapeutic influence on signaling pathways associated with neurological disorders.

### 5.3 Neuroinflammation modulation

Numerous neurological disorders are influenced by neuroinflammation, which plays a crucial role in neurological development. Exosomes produced from stem cells are included in ncRNAs, which can target inflammatory pathways and control the production of pro- or anti-inflammatory proteins. By modulating neuroinflammatory responses, these ncRNAs can reduce inflammation (Zhang Z. et al., 2021; Zhang Y. et al., 2021), attenuate glial activation, and create a more favorable environment for neuronal survival (El Bassit et al., 2017; Pan et al., 2020; Liu J. et al., 2022) and regeneration (Zhang Y. et al., 2021; Pan et al., 2021; Huang et al., 2021; Patel et al., 2018). Below is a summary of the effect of ncRNAs derived from exosomes originating from stem cells on neuroinflammation and the implications for neurological disorders:

#### 5.3.1 Regulation of inflammatory responses

Exosomes originating from stem cells possess the capacity to transport specific types of ncRNAs, such as miRNAs and lncRNAs, which selectively target genes associated with immune responses and inflammation (Zhang Z. et al., 2021; Tian et al., 2022; Zhang Y. et al., 2021). These exosomal ncRNAs can regulate the levels of inflammatory substances in the body, such as cytokines (such as interleukins and tumor necrosis factor-alpha) and chemokine (Tian et al., 2022). They also affect the production of mediator-producing enzymes, such as cyclooxygenases and inducible nitric oxide synthases. By downregulating the expression of these pro-inflammatory molecules (Jiang and Zhang, 2021; Shao et al., 2020; Dong et al., 2022), exosomal ncRNAs can attenuate neuroinflammation in neurological disorders.

#### 5.3.2 Regulation of glial activation

Glial cells, such as microglia and astrocytes, have functions in processes related to inflammation in the brain. Stem cell-derived exosomes can carry ncRNAs that target genes involved in the activation and polarization of glial cells. For instance, specific exosomal miRNAs can control the activation of microglia, causing them to transition from an inflammatory (M1) state to an anti-inflammatory (M2) state (Chang et al., 2021; Shao et al., 2020; Dong et al., 2022; Yang et al., 2022; Zhao et al., 2020). By modulating the activation and polarization of glial cells (Zhang Z. et al., 2021; Xiaoying et al., 2020), the ncRNAs contained within stem cell-derived exosomes can regulate the immune responses and help reduce neuroinflammation.

#### 5.3.3 Modulation of neuroinflammatory signaling pathways

Neuroinflammation involves he activation of pathways such as the IRAK1/TRAF6 signaling pathway (Zhang Z. et al., 2021). Stem cell-derived exosomes can transport ncRNAs that target components of these pathways, thereby affecting their activity and downstream signaling. By modulating the activity of these signaling pathways, exosomal ncRNAs can influence the expression of pro-inflammatory genes and modulate neuroinflammatory responses (Zhang Z. et al., 2021).

#### 5.3.4 Regulation of immune cell function

Immune cells, including microglia and infiltrating immune cells, play critical roles in neuroinflammation. Exosomes produced from stem cells can deliver ncRNAs to cells of the immune system, thereby modulating their function and behavior. For instance, exosomal ncRNAs can influence the phenotype and activation state of microglia (Zhang Z. et al., 2021; Xiaoying et al., 2020), and also regulate the recruitment and activation of peripheral immune cells. By modulating immune cell function, exosomal ncRNAs can influence neuroinflammatory processes in neurological disorders (Zhang Z. et al., 2021; Xiaoying et al., 2020).

### 5.4 Intercellular communication and paracrine effects

**FIGURE 5:**
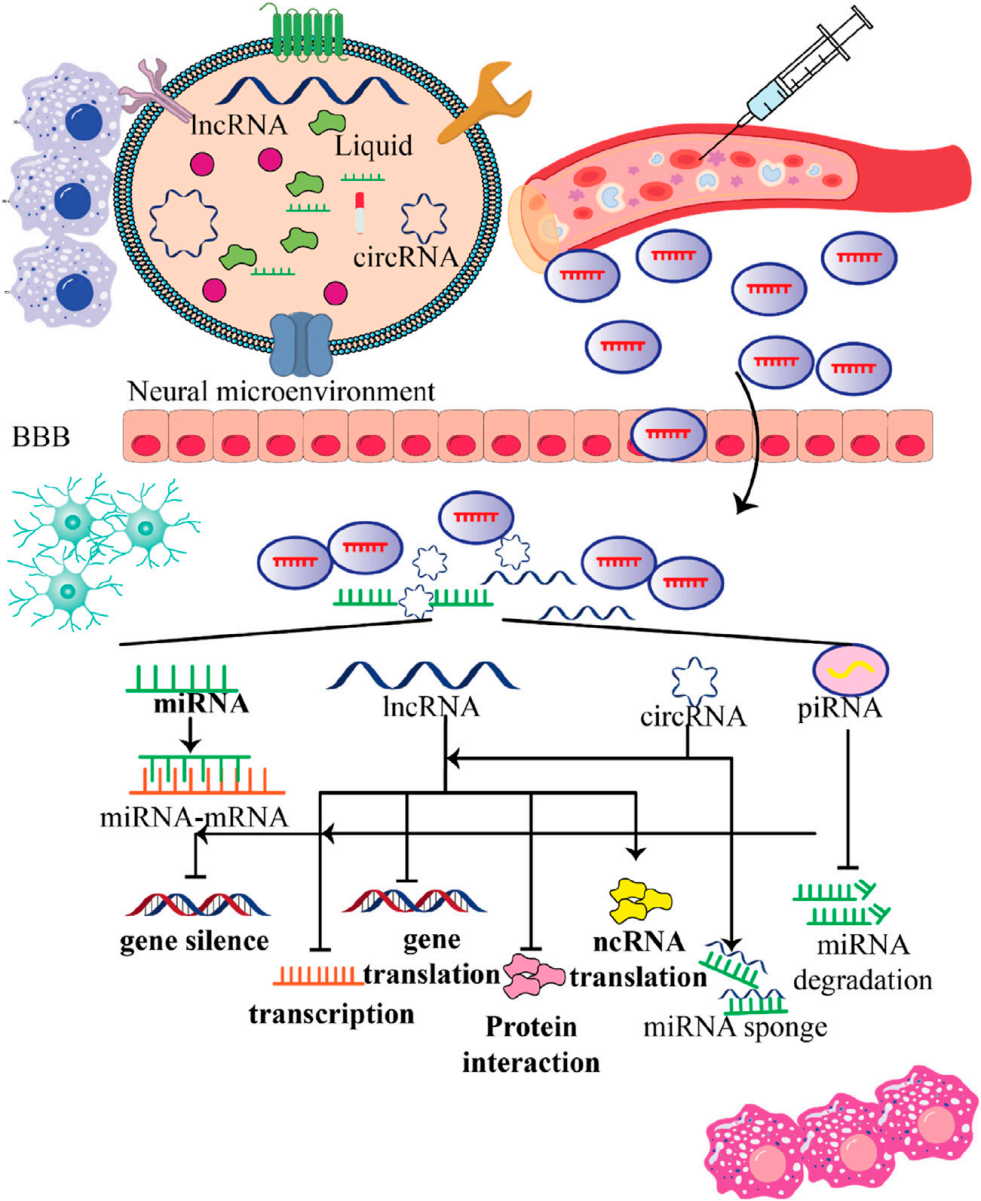
Various functions stem cell-produced exosomes contain ncRNAs within the recipient cells. This figure emphasizes the multifunctional roles of ncRNAs contained in stem cell-derived exosomes within the neural microenvironment. By crossing the blood-brain barrier (BBB) and influencing gene expression in recipient cells, these exosomes hold significant potential for therapeutic applications in treating nervous system disorders, highlighting their importance in innovative medical strategies.

NcRNAs found in stem cell-produced exosomes can be absorbed by recipient cells, thereby changing the function of those cells. By transferring their content, these exosomes can regulate gene expression, signaling pathways, and various tasks within the recipient cells (Figure 5). This communication between cells can have effects and contribute to therapeutic benefits for nearby cells and tissues. Now let’s take a look at how ncRNAs from stem cell-derived exosomes impact communication and explore their potential implications for neurological disorders.

#### 5.4.1 Transfer of ncRNAs

Exosomes obtained from stem cells have the capability to transport various categories of ncRNAs, including miRNAs, lncRNAs, and circRNAs. The nervous system’s recipient cells, such as endothelial cells (Wang et al., 2022; Hill, 2019; Venkat et al., 2018), microglia (Chang et al., 2021; Shao et al., 2020; Dong et al., 2022; Yang et al., 2022; Zhao et al., 2020), neurons (Zhuang et al., 2023; Pan et al., 2021; Zhao Y. et al., 2022; Liu Y. et al., 2022), and astrocytes (Huang W. et al., 2023), can receive these ncRNAs from stem cells. These exosomal ncRNAs can influence gene expression and cellular function in the recipient cells that absorb the exosomes.

#### 5.4.2 Regulation of gene expression

Stem cell-derived exosomal ncRNAs can regulate gene expression in recipient cells through mechanisms. For instance, miRNAs have the ability to bind to mRNAs and cause translation inhibition or mRNA destruction. LncRNAs can change gene expression at both the transcriptional and non-transcriptional levels by interacting with proteins, RNA, or DNA. Through these actions that change gene expression patterns, exosomal ncRNAs have the potential to impact processes relevant to neurological disorders, including neuroinflammation, neurogenesis (Zhang Y. et al., 2021; Pan et al., 2021), synaptic plasticity, and neuronal survival (El Bassit et al., 2017; Pan et al., 2020; Liu J. et al., 2022).

#### 5.4.3 Modulation of cellular phenotypes

Extracellular ncRNAs can influence the characteristics of cells by controlling their development, growth, and overall functions. For instance, ncRNAs found in exosomes from stem cells can support the transformation of progenitor cells into neurons, improve survival rates (El Bassit et al., 2017; Pan et al., 2020; Liu J. et al., 2022), guide microglia toward an anti-inflammatory state (Chang et al., 2021; Shao et al., 2020; Dong et al., 2022; Yang et al., 2022; Zhao et al., 2020), or stimulate angiogenesis (Bai et al., 2022; Zhang Y. et al., 2021; Wang et al., 2022; Jiang et al., 2021). These actions play a role in facilitating tissue healing, protecting the system, and promoting functional restoration in neurological disorders.

#### 5.4.4 Regulation of signaling pathways

Exosomes, which are membrane-bound vesicles, contain ncRNAs that can impact the behavior of recipient cells by affecting various cellular processes. One interesting example is how stem cell-derived exosomal miRNAs can interact with components of signaling pathways like Wnt/β (Xu et al., 2019), mTOR (Wei et al., 2020; Huang W. et al., 2023), NF-κB (Zhang Z. et al., 2021; Zhuang et al., 2023; Jiang and Zhang, 2021; Sun and Xu, 2023), NGF/TrkA (Wang et al., 2021), PI3K/Akt (Pan et al., 2020), or AKT/ERK (Jiang et al., 2021) pathways. This interaction can then influence responses related to synaptic plasticity and neuroinflammation. By modulating signaling pathways in the recipient cells, the stem cell-derived exosomal ncRNAs play a role in regulating various cellular functions. This modulation has important implications for understanding and potentially treating neurological disorders.

#### 5.4.5 Neuroprotection and tissue repair

Exosomes produced from stem cells can convey ncRNAs that are essential for enhancing neuroprotection and expediting tissue restoration (Liu J. et al., 2022; Chang et al., 2021; Shao et al., 2020) in various neurological disorders. The presence of stem cell-derived exosomal ncRNAs enhances cell survival (El Bassit et al., 2017; Pan et al., 2020; Liu J. et al., 2022), reduces stress (Tian et al., 2022; Wang Q. S. et al., 2023), regulates inflammation, stimulates the formation of new blood vessels (Bai et al., 2022; Zhang Y. et al., 2021; Wang et al., 2022; Jiang et al., 2021), and facilitates tissue remodeling. These beneficial effects contribute to the preservation of function, restoration of damaged tissue, and overall promotion of recovery in individuals with disorders. The functional mechanisms of ncRNAs can vary depending on the specific type of ncRNAs they interact with and the particular neurological disorder being investigated. More research is necessary to understand the mechanisms through which ncRNAs from exosomes derived from stem cells confer therapeutic benefits. In addition, optimizing delivery methods for use requires exploration.

#### 5.4.6 NcRNAs from stem cell-derived exosome and neurological disorders

NcRNAs obtained from exosomes derived from stem cells have shown encouraging prospects concerning neurological disorders. These ncRNAs have the capability to regulate gene expression and manipulate cellular processes in the cells to which they are transferred, presenting potential therapeutic advantages. Because they can alter gene expression and neuroinflammation (Zhang Z. et al., 2021; Zhang Y. et al., 2021), ncRNAs produced from stem cell-derived exosomes have therapeutic potential in neurological diseases (Zhang Z. et al., 2021; Zhang Y. et al., 2021; Elballal et al., 2024), neurodegenerative diseases (Zhang Y. et al., 2021; Pan et al., 2021; Huang et al., 2021; Patel et al., 2018), and synaptic plasticity (Pan et al., 2020; Hu and Li, 2017). By targeting specific molecular pathways and cellular processes, these ncRNAs can exert neuroprotective effects (Xie et al., 2023; Pan et al., 2020; Zhuang et al., 2023; Luo et al., 2022; Losurdo et al., 2020), enhance functional recovery, and slow disease progression. To fully understand the mechanisms of action, optimize delivery methods, and ascertain the effectiveness and safety of stem cell-derived exosomes in human patients, more investigation and clinical trials are still needed. We present a few examples of neurological disorders associated with the involvement of ncRNAs derived from stem cell exosomes (Zhang Z. et al., 2021; El Bassit et al., 2017; Tian et al., 2022; Bai et al., 2022; Xie et al., 2023; Pan et al., 2020; Zhuang et al., 2023; Wang et al., 2021; Zhang Y. et al., 2021; Pan et al., 2021; Liu J. et al., 2022; Chang et al., 2021; Luo et al., 2022; Wang et al., 2022; Xu et al., 2019; Jiang et al., 2021; Wei et al., 2020; Jiang and Zhang, 2021; Sun and Xu, 2023; Fan et al., 2021; Huang et al., 2021; Patel et al., 2018; Shao et al., 2020; Dong et al., 2022; Yang et al., 2022; Zhao et al., 2020; Xiaoying et al., 2020; Zhao Y. et al., 2022; Liu Y. et al., 2022; Huang W. et al., 2023; Wang Q. S. et al., 2023; Li et al., 2022; Liu B. et al., 2022; Wang Y. et al., 2023; Li et al., 2020; Qian et al., 2022; Shao et al., 2023; Yu et al., 2019; Ji et al., 2019; Zhang and Han, 2022; Shao et al., 2022; Wang et al., 2020) (Table 1).

**TABLE 1:** Various ncRNAs in different neurological diseases.

Non-coding RNAs	Stem cell-derived exosome	Type of neurological disorders	Target	Ref
miR-150-5p	BMMSCs	Cerebral ischemia/reperfusion injury	TLR5	Li et al. (2022)
miR-455-5p	BMMSCs	Spinal cord ischemia-reperfusion injury	Nogo-A	Liu Y. et al. (2022)
miR-193b-5p	BMMSCs	Ischemic stroke	AIM2	Wang Y. et al. (2023)
miR-124	MSCs	Oxygen-glucose deprivation/reperfusion (OGD/R) injury	GLT-1/mTOR	Huang Y. et al. (2023)
mirR-124-3p	BMMSCs	SCIRI	Ern1 and promoting M2 macrophage polarization	Li et al. (2020)
miR-124-3p	BMSCs	Traumatic brain injury	p38 MAPK/GLT-1	Zhuang et al. (2023)
miR-124-3p	NSCs	Glioma	FLOT2/AKT1	Qian et al. (2022)
miR-137	BMMSCs	Spinal Cord Injury	-	Shao et al. (2023)
miR-125a	BMMSCs	Spinal cord injury	IRF5/promotes M2 macrophage polarization	Chang et al. (2021)
miR-23a-3p	MSCs	Cerebral infarction	pro-inflammatory and anti-inflammatory signaling pathways	Dong et al. (2022)
miR-150-3p	MSCs	Intracerebral hemorrhage (ICH)	TRAF6/NF-κB	Sun and Xu (2023)
miR-150-3p	NSCs	Hypoxic-ischemic brain injury	CASP2	Luo et al. (2022)
miR −133b	MSCs	Glioma	Wnt/β-catenin/EZH2	Xu et al. (2019)
miR-146a	MSCs	Diabetic peripheral neuropathy	-	Fan et al. (2021)
miR-146a-5p	MSCs	Ischemic stroke	IRAK1/TRAF6/NF-κB signaling	Zhang Y. et al. (2021)
miR-199a	MSCs	Glioma	AGAP2	Yu et al. (2019)
miR-223	MSCs	Alzheimer’s diseases	PTEN- PI3K/Akt/mTOR pathway	Wei et al. (2020)
miR-223-3p	MSCs	Cerebral ischemia	M1 polarization	Zhao et al. (2020)
miR-21	MSCs	Spinal cord injury	-	Ji et al. (2019)
miR-21-5p	USCs	Rett syndrome	EPha4/TEK	Pan et al. (2021)
miR-132-3p	MSCs	Brain ischemic injury	Ras/PI3K/Akt/eNOS signaling	Pan et al. (2020)
miR-145-5p	MSCs	Spinal cord injury	TLR4/NF-κB signaling	Jiang and Zhang (2021)
miR-199a-3p/145-5p	UMSCs	Traumatic spinal cord injury	NGF/TrkA signaling	Wang et al. (2021)
miR-374-5p	NSCs	Spinal cord injury	STK-4	Zhang and Han (2022)
miR-494	MSCs	Spinal Cord Injury	-	Huang et al. (2021)
miR-944	GSCs	Glioma	AKT/ERK signaling	Jiang et al. (2021)
miR-17–92	BMMSCs	Traumatic Brain Injury	-	Zhang Z. et al. (2021)
LncRNA Gm36569	MSCs	Acute spinal cord injury	FSP1 miR-5627-5p/FSP1	Shao et al. (2022)
LncRNA KLF3-AS1	BMSCs	Cerebral ischemia/reperfusion injury	miR-206/USP22 axis	Xie et al. (2023)
LncRNA MALAT1	hASCs	Traumatic brain injury	-	Patel et al. (2018)
LncRNA MALAT1	hASCs	Brain injury	PKCδII	El Bassit et al. (2017)
LncRNA PTENP1	MSCs	Spinal cord injury	miR‐21/miR‐19b/PTEN	Wang et al. (2020)
LncRNA Gm37494	ADSCs	Spinal Cord Injury	Shifting Microglial M1/M2 Polarization	Shao et al. (2020)
LncRNA TCTN2	MSCs	Spinal Cord Injury	miR-329-3p/IGF1R	Liu B. et al. (2022)
LncRNA H19	NSCs	Cerebral Ischemic Stroke	miR-18a/VEGF	Wang et al. (2022)
LncRNA ZFAS1	BMSCs	Ischemic stroke	miRNA-15a-5p	Wang Y. et al. (2023)
circZFHX3	MSCs	Spinal cord injury	miR-16-5p-IGF-1	Tian et al. (2022)
circ-Rps5	ADSCs	AIS-induced brain injury	miR-124-3p- SIRT7	Yang et al. (2022)
circ_003564	BMSCs	Spinal Cord Injury	Reduction of pyroptosis markers	Zhao Y. et al. (2022)
circFUNDC1	HBMECs	Ischemic stroke	miR-375-PTEN	Bai et al. (2022)
CircOGDH	NSCs	AIS	miR-5112- COL4A4	Liu J.et al. (2022)
CircHivep2	ADSCs	Epilepsy	miR-181a-5p- SOCS2	Xiaoying et al. (2020)

## 6 Alzheimer’s disease

A disorder known as Alzheimer’s disease (AD) affects the brain and causes deposits of materials known as neurofibrillary tangles and amyloid beta plaques (Scheltens et al., 2021). The possibility of using stem cell-derived exosomes and their contents to help treat AD has been investigated by several researchers. Liu J. et al. (2022) discovered that in an AD model, there was an increase in HIF-1α expression, apoptosis, and a decrease in miR-223. Exosomes generated from MSCs were discovered to be taken up by AD cells, leading to enhanced cell motility, reduced apoptosis, and elevated miR-223 levels. The use of KC7F2, a hypoxia inhibitor, restored these effects. Additional research showed that exosomal miR-223 produced from MSCs inhibited cell death by targeting PTEN, a protein that controls the PI3K/Akt signaling pathway (Wei et al., 2020). The activation of this pathway plays a protective role against apoptosis. Moreover, exosomes isolated from the blood serum of AD patients, stimulated cell death. According to this study, exosomal miR-223, which is produced from MSCs, influence cells by controlling apoptosis via the PTEN PI3K/Akt pathway. The findings of this study provide insights into a therapeutic approach involving MSC-derived exosomes and miR-223 for AD treatment (Wei et al., 2020).

## 7 Spinal cord injury

Spinal cord injury (SCI) causes damage to the spinal cord, which results in the loss of motor and sensory functions (Eli et al., 2021). Promising results have been obtained using stem cell-derived exosomes carrying ncRNAs in that promote functional recovery after SCI (Liu et al., 2021; Jendelova, 2018). In SCI models, exosomes loaded with ncRNAs can enhance neuronal survival (Liu J. et al., 2022), reduce inflammation, and facilitate axonal regeneration (Zhang Y. et al., 2021; Pan et al., 2021; Huang et al., 2021; Patel et al., 2018). In this study, we present examples of ncRNAs derived from different stem cells that exhibit therapeutic effects in animal models of SCI.

### 7.1 miR-455-5p

The potential use of modified lentivirus-containing miR-455-5p-containing exosomes derived from bone marrow mesenchymal stem cells (BMSCs) in spinal cord ischemia reperfusion (SCIR) damage cases was investigated by Patel et al. (2018). Following SCIR, a decrease in miR-455-5p expression was observed, indicating its role in damage. After separation from BMSCs, exosomes containing miR-455-5p were injected into an SCIR rat model. When miR-455-5p was administrated to SCIR rats, hind limb motor function scores showed improvement in hind limb recovery. Histopathological analysis using HE and Nissl staining demonstrated that exosomal miR-455-5p helped reduce the abnormalities observed in the injured cord. Moreover, exosomes containing miR-455-5p helped to lower apoptosis and activation of autophagy following SCIR. The study also noted a decrease in the expression of Nogo-A within the spinal cord tissues treated with exosomes containing miR-455-5p. Nogo-A is a protein that is directly targeted by miR-455-5p. These findings suggest that miR-455-5p may regulate Nogo-A expression after SCIR injuries, emphasizing the benefits of exosomes that contain this miRNA in terms of inducing autophagy and preventing apoptosis. This research helps enable a better understanding of the therapeutic function of administering exosomes containing miR-455-5p to patients with SCIR (Liu B. et al., 2022).

### 7.2 miR-137

The effects of bone marrow mesenchymal stromal cells (BMMSCs) generated from exosomes expressing miR-137 on SCI recovery were investigated by Shao et al. (2020). Rats with SCI and a negative control mimic were used in this investigation. Reduced tissue damage, enhanced locomotor capability viability, decreased cytokine production, and decreased miR-137 expression in the spinal cord tissue. According to the study, miR-137 overexpressing BMMSC exosomes may be a functional therapeutic approach for the recovery of SCI (Shao et al., 2023).

### 7.3 miR-125a

In case of SCI, exosomes produced from BMMSCs can support neuroprotection and macrophage polarization. Chang et al. (2021) used a rat model and showed that BMMSCs-Exo treatment significantly enhanced locomotor function recovery in SCI rats, decreased apoptosis and degeneration, and led to macrophages adopting the M2 phenotype, associated with inflammatory responses and tissue repair capabilities. The decrease in the expression of interferon factor 5 (IRF5) was also observed to contribute to the promotion of M2 macrophage polarization and the mitigation of inflammation. The researchers found a connection between miR-125a, a miRNA found in BMMSCs-exo particles, and IRF5, which directly targets and negatively regulates IRF5 expression. Inhibiting miR-125a in BMMSCs-exo cells increased IRF5 expression in cord tissues, thereby weakening its effects against SCI (Chang et al., 2021).

### 7.4 miR-21

The involvement of miR-21 in SCI processes has been established. It was observed that miR-21 exhibits properties against SCI. However, the impact of exosomes obtained from rat MSCs on SCI and the specific role miR-21 in these effects are not fully understood. Ji et al. (2019) conducted investigations to explore the potential impact of miR-21 and exosomes derived from rat MSCs on SCI and their potential therapeutic benefits. In this study, the researchers analyzed the influence of exosomes derived from rat MSCs on SCI. The researchers specifically focused on examining the role miR-21 concerning these effects. The study’s results showed that when exosomes were taken from rat’s MSCs, they could not protect against SCI due to low levels of miR-21. However, when there was an increase in miR-21 expression in the MSCs of rats, the protective effects of exosomes derived from these cells were restored. The research also found that the MSCs derived from rats exhibit insulin resistance, which in turn results in decreased levels of miR-21 being packaged into the exosomes secreted by these cells. Based on their findings, Ji et al. (2019) proposed that the lack of miR-21 in MSCs from rats plays a role in diminishing the beneficial effects of exosomes derived from these cells when used to treat spinal cord injuries. This study emphasizes the potential of targeting miR-21 as a strategy for addressing cord injuries (Ji et al., 2019).

### 7.5 miR-145-5p

Jiang and Zhang (2021) showed that MSCs containing miR-145-5p can reduce inflammation in SCI by influencing the TLR4/NF-κB signaling pathway. MSCs have shown potential as treatment options for SCI due to their ability to regenerate and modulate the system. This study found that administering exosomes containing miR-145-5p decreased the release of pro-inflammatory cytokines and suppressed the activation of the TLR4/NF-κB signaling pathway. In rats, the researchers found that rats treated with miR-145-5p-containing exosomes experienced decreased inflammation and enhanced recovery compared to the control group. These findings suggest that exosomes derived from MSCs can potentially facilitate tissue restoration and promote recovery in cases of spinal cord injury (Jiang and Zhang, 2021).

### 7.6 miR-374-5p

Zhang and Han (2022) reported that exosomes derived from NSCs can help alleviate spinal cord injury by promoting autophagy and suppressing apoptosis. The exosomes contain miR-374-5p helps reduce cell death in damaged nerve cells by stimulating the cellular process of autophagy. These researchers also identified a gene called STK 4, which is controlled by miR-374- 5p and contributes to the function of exosomes. The increased levels of miR-374-5p in nerve cell exosomes can improve recovery from cord injuries by triggering autophagy. These findings offer insights into the potential use of from NSC-derived exosomes for spinal cord injury treatment (Zhang and Han, 2022).

### 7.7 miR-494

Huang et al. (2021) studied the potential of exosomes modified with miR-494, a gene responsible for regulating spinal cord injury, for promoting recovery from SCI. The exosomal miR-494 was found to have beneficial effects, including inhibiting inflammation, reducing apoptosis, and facilitating the regeneration of neurofilaments in nerve cells. Experiments were conducted on living organisms and in the laboratory. Pishavar et al. (2022) showed that Exo-miR-494 reduced inflammation, protected cells, and enhanced the production of anti-inflammatory molecules. Moreover, it promoted neurofilaments regeneration and improved behavioral function recovery in SCI patients (Huang et al., 2021).

### 7.8 miR-199a-3p/145-5p

Wang et al. (2021) studied the potential of exosomes containing miR-199a-3p/145-5p from human umbilical cord mesenchymal stem cells (hUCMSCs) for treating SCI. The study found that suppressing the expression of miR-199a-3p and miR-145-5p can enhance the differentiation of PC12 cells *in vitro* when they are treated with lipopolysaccharide (LPS). This effect is mediated by the modulation of the NGF/TrkA signaling pathway. The researchers also found that miR-199a-3p targets Cblb and Cbl, which decreases TrkA ubiquitination levels, activating the Akt and Erk pathways involved in NGF/TrkA signaling. Conversely, when Cblb and Cbl are overexpressed, TrkA ubiquitination increases, deactivating the Akt and Erk pathways. These researchers used Western blot and immunoprecipitation techniques to confirm the interaction between the proteins TrkA and Cblb, as well as the interaction between TrkA and Cbl. In an experiment in rats with SCI, exosomal miR-199a-3p/145-5p increased TrkA expression at the injury site and enhanced locomotor function (Wang et al., 2021).

### 7.9 miR-124-3p

Yang et al. (2020) found that miR-124-3p, obtained from exosomes from BMMSCs, can potentially reduce spinal cord ischemia reperfusion injury (SCIRI), a condition that can lead to paralysis and neural dysfunction. Researchers showed that Ern1 is highly expressed in SCIRI, whereas M2 polarization markers are poorly expressed. Silencing Ern1 increased the expression of M2 polarization markers. miR-124-3p targets and negatively regulates Ern1, enhancing M2 macrophage polarization. Injection of miR-124-3p-containing exosomes into SCIRI rats reduced cell apoptosis, improved tissue condition, and attenuated nerve injury. These researchers concluded that miR-124-3p derived from BMMSC-derived exosomes can reduce SCIRI and associated nerve injury by inhibiting Ern1 and promoting M2 macrophage polarization (Yang et al., 2020).

### 7.10 lncRNA Gm36569

Shao et al. (2022) reported that exosomes from MSCs can treat acute spinal cord injury (ASCI). The researchers created mouse and a cell model for hypoxia in order to study the effects of MSCs and MSC-derived exosomes. The results illustrated that MSCs and MSCs-Exos reduced oxygen species and ferrous iron production, increased ferroptosis suppressor FSP1 expression, and improved function in mice with ASCI. They also reduced hypoxia-induced neuronal cell ferroptosis and promoted cell growth. This study found that MSCs-Exos containing lncGm36569 could inhibit neuronal cell ferroptosis by modulating the miR-5627-5p/FSP1 axis, contributing to the attenuation of neuronal dysfunction in ASCI cases. These findings may help improve the function of mice with ASCI (Shao et al., 2022).

### 7.11 lncRNA PTENP1

Wang et al. (2020) reported that PTENP1 plays a crucial role in the healing process of SCI. MSCs modified with PTENP1 shRNA were used as indicators of SCI treatment. The shRNA reduced PTENP1 and PTEN levels while increasing miR-21 and miR-19b levels. PTEN expression and apoptosis were highest in the SCI group but were reduced to a similar level after treatment with exosomes plus PTENP1-shRNA. The result suggested that PTENP1 regulates the expression of miR-19b and miR-21, suggesting that exosomes derived from PTENP1-shRNA-transfected cells could be a novel biomarker for SCI treatment (Wang et al., 2020).

### 7.12 lncRNA Gm37494

Researchers have found that exosomes from fat tissue-based mesenchymal stem/stromal cells (ADSCs) can treat spinal cord injuries. Shao et al. (2020) recommended that exosomes released under high-oxygen conditions are more effective in repairing SCI by reducing inflammatory factors, promoting functional recovery, and shifting microglial polarization from the M1 pro-inflammatory phenotype to the M2 anti-inflammatory phenotype. The expression of the gene lncGm37494 was significantly higher in exosomes secreted by ADSC under hypoxia (HExos) than in Exos cells. Increasing lncGm37494 levels led to cell polarization into M1/M2 states by suppressing miR-130b-3p and enhancing PPARγ expression. Exosomes derived from ADSCs genetically modified to overexpress lncGm37494 showed the same effects as naturally occurring HExos in treating SCI. The result suggest that HExos carry lncGm37494, which regulates the microenvironment and may hold promise for SCI treatment (Shao et al., 2020).

### 7.13 lncRNA TCTN2

Liu J. et al. (2022) showed that exosomes from MSCs modified with a ncRNA called TCTN2 can improve the healing process in cord injuries by regulating the miR-329/3p/IGF1R pathway. The genetic modification of MSCs affects their ability to produce exosomes, and these exosomes contain components like the miR-329-3p. This miRNA targets and regulates the activity of IGF1R, which is involved in cell growth and survival. By delivering the miR-329-3p to the site of the spinal cord injury through exosomes, it helps control and regulate the expression and function of the IGF1R protein. The modulation of the miR-329-3p/IGF1R axis may contribute to reduced inflammation, enhanced neuronal survival, and improved tissue repair in injured spinal cords (Liu J. et al., 2022).

### 7.14 circZFHX3

Tian et al. (2022) found that exosomal circZFHX3 can help mitigate LPS-induced injury in MSCs in the context of SCI. They found that in mice with SCI, circZFHX3 and IGF-1 levels decreased, whereas miR-16 5p levels increased. The study found that the exosomal circZFHX3 enhanced cell viability and reduced apoptosis, inflammation, and oxidative stress in BV-2 cells that were treated with LPS. The underlying mechanism circZFHX3 acting as a sponge for miR-16-5p, regulating IGF 1 expression. In a mouse model of SCI, exosomal circZFHX3 also alleviated cell injury. These findings suggest that exosomal circZFHX3 is a potential therapeutic strategy for SCI treatment (Tian et al., 2022).

### 7.15 circ_003564

Zhao Y. et al. (2022) investigated the role of exosome-delivered circRNAs profiles in the therapeutic potential of BMSCs for SCI. Rat primary neurons were cultured under different conditions, including standard medium, BMSCs, BMSCs + GW4869, and BMSC-derived exosomes. The present study showed that BMSCs and BMSC-derived exosomes reduced inflammasome-related pyroptosis markers in H2O2-treated neurons. However, exosome-free BMSCs did not significantly reduce these factors. The most effective circRNAs was circ_003564, which was knocked down in BMSC-derived exosomes. *In vivo* experiments have shown that BMSC-derived exosomes improve functional recovery, reduce tissue injury, and decrease inflammasome-related pyroptosis in SCI rats (Zhao Y. et al., 2022).

## 8 Stroke

A stroke is a type of brain event that can cause damage to the system. Researchers have explored using exosomes derived from stem cells, which are packed with ncRNAs, as a possible treatment approach for stroke. These exosomes, containing ncRNAs, have their ability to protect neurons from dying, stimulate angiogenesis, and improve functional recovery in experimental models of ischemic stroke.

### 8.1 miR-193b-5p

Researchers have identified a therapeutic mechanism for ischemic stroke using BMSC-derived exosomal miR-193b-5p. Temple (2023) found that miR-193b-5p directly binds to the 3′-untranslated region of absent in melanoma 2 (AIM2) gene, suggesting a regulatory relationship between miR-193b-5p and AIM2. Overexpression of BMSCs exosomes exhibited improved cell health, lower toxicity, reduced levels of AIM2, Gasdermin D N-terminal fragment (GSDMD N), and cleaved caspase 1, and decreased production of IL-1β and IL-18, markers of pyroptosis. In a middle cerebral artery occlusion (MCAO) model, miR-193b-5p-BMSC-Exos had more significant effects than normal exosomes, including decreased levels of pyroptosis-related molecules and reduced infarct volume, indicating a reduction in cerebral ischemia/reperfusion injury. Delivering miR-193b-5p through these exosomes could represent an approach for treating ischemic stroke by influencing pyroptosis-related pathways (Temple, 2023).

### 8.2 miR-146a-5p

Researchers have explored the therapeutic potential of exosomes derived from human umbilical cord mesenchymal stem cells (hUMSCs) in the treatment of The researchers found that hUMSC-derived exosomes contain a specific miRNA called miR-146a-5p, and this miRNA. Ischemic stroke is a condition characterized by reduced blood flow to the brain, leading to tissue damage and neuroinflammation. Zhang Z. et al. (2021) found that hUMSC-derived exosomes could access damaged tissues *in vivo* and *in vitro*. They also observed a decrease in inflammation caused by microglia. The study also tested a brain ischemia model on live subjects and showed reductions in infarct volume, improvement in neurological deficits, and a decrease in microglial activation 3 days after the ischemic incident. The researchers found that hUMSC-derived exosomes contain a specific miRNA called miR-146a-5p, and this miRNA was shown to play a role in mediating the therapeutic effects observed in their study. When partially suppressed, miR-146a-5p reversed some of the beneficial effects of the exosomes, indicating its involvement in reducing neuroinflammation. The researchers proposed that miR-146a-5p derived from hUMSC exosomes suppresses the IRAK1/TRAF6 signaling pathway, which is known to play a role in neuroinflammatory responses (Zhang Z. et al., 2021). Although the beneficial role of miR-146a-5p in this study is very promising, there are contradictory results in other neurodegenerative diseases such as AD. The role of miR-146a in AD and its related neuroinflammation is indeed complex and somewhat controversial. miR-146a primarily functions by inhibiting Toll-like receptor (TLR) signaling pathways. This inhibition is crucial because TLRs are often implicated in the inflammatory responses associated with AD. By modulating TLR signaling, overexpressed miR-146a can help reduce neuroinflammation. miR-146a plays a role in regulating the activation of glial cells, which are involved in the immune response in the brain. miR-146a may help mitigate the accumulation of Aβ plaques, a hallmark of AD. miR-146a may also influence tau protein modifications, which are critical in AD pathology (Mai et al., 2019; Li et al., 2011). The overall effect of these processes suggests that miR-146a could rescue cognitive functions in AD models, providing a potential therapeutic target. While many studies indicate that overexpressed miR-146a can reduce neuroinflammation and improve cognitive function in AD models, this is not the whole story. Evidence suggests that miR-146a may inhibit complement factor H (CFH), which is crucial for regulating the complement system and preventing excessive inflammation. By downregulating CFH, miR-146a could potentially exacerbate proinflammatory responses in neurons, particularly *in vitro* (Lukiw et al., 2008; Pogue et al., 2009). Therefore, by addressing these areas, researchers can provide clearer insights into the dual roles of miR-146a and its potential as a therapeutic target in neurological diseases.

### 8.3 miR-23a-3p

Dong et al. (2022) explored the potential therapeutic application of MSCs-Exos for treating cerebral infarction (CI). They revealed that MSCs-Exos from human umbilical cord blood exhibited elevated miR-23a-3p levels, which significantly influenced the activation and polarization of microglia. The study found that exosomes derived from BMSCs improved neuronal function and reduced the size of the brain infarction in a rat model of MCAO. However, upon knocking down miR-23a-3p, the therapeutic effects of BMSCs-Exos on CI were reversed. The knockdown of miR-23a-3p limited the inhibition of microglial activation and M1 polarization induced by BMSCs-Exos in response to MCAO and reduced pro-inflammatory factors (Dong et al., 2022).

### 8.4 miR-150-3p

Exosomes containing miR-150-3p from mouse bone marrow mesenchymal stem cells have been shown by Sun and Xu (2023) to be an efficient treatment for hemorrhage or bleeding caused by blood vessel rupture. They showed that miR-150-3p expression is lower in ICH-infected brain tissue and interacts with TRAF6. They suggested inhibiting miR-150-3p may affect ICH injury through the TRAF6/NLRP3 axis. The present study also examined how MSC-derived exosomal miR-150-3p effects the gut microbiota and metabolism. Faecal microbiota transplantation (FMT) can help understand the impact of gut microbiota on ICH (Sun and Xu, 2023).

### 8.5 lncRNA ZFAS1

Researchers conducted a study to investigate the effects of exosomes derived from BMSCs on stress and inflammation in stroke patients. Wang Q. S. et al. (2023) studied the impact of exosomes on BV-2 cells’ proliferation, inflammation, oxidative stress, and apoptosis in scenarios of oxygen-glucose deprivation/reperfusion and middle cerebral artery occlusion. The study also examined the interaction of RNA called ZFAS1 with miR-15a-5p. They showed that co-culturing BV-2 cells with BMSCs increased cell growth, reduced cytokine levels, and reduced oxidative stress markers. However, inhibiting exosome secretion reversed these effects. ZFAS1 regulates miR-15a-5p expression, promoting cell proliferation, and reducing oxidative stress. Overexpression of ZFAS1 diminishes the protective effects of exosomal ZFAS1. Treatment with exosome-overexpressing reduces oxidative stress, CI, and inflammation in MCAO mice (Wang Q. S. et al., 2023).

### 8.6 lncRNA H19

Exosomes carrying H19 disrupt the BBB in ischemic stroke due to the interaction between H19, miR-18a, and VEGF (Wang et al., 2022). The disrupted BBB, which typically protects the brain from harmful substances, can potentially worsen brain damage. H19, which is carried by exosomes, interacts with miR-18a, inhibiting its expression, which impacts VEGF’s role in angiogenesis and blood vessel permeability. The H19/miR-18a/VEGF axis induces BBB disruption in cerebral ischemic stroke, leading to increased VEGF levels, vascular permeability, and BBB breakdown. This disruption can lead to the entry of inflammatory cells and molecules into the brain, potentially exacerbated by ischemic stroke (Wang et al., 2022).

### 8.7 circ-Rps5

Researchers discovered that exosomes from hypoxic pre-treated adipose-derived stem cells (hASCs) can enhance cognitive function and mitigate neuronal damage in the post-stroke hippocampus. Yang et al. (2022) demonstrated that exosomes delivered with circ-Rps5 influenced SIRT7 and miR-124-3p, leading to microglia transitioning from M1 phenotype to M2 phenotype in the presence of LPS. ADSC exosomes modified with circ-Rps5 enhanced cognitive function by reducing neuronal damage and promoting M2 microglia/macrophage polarization in the hippocampus. This study suggests that ADSC exosomes can attenuate acute ischemic stroke (AIS)-induced brain injury (Yang et al., 2022). Of course, ischemic conditions lead to an accumulation of reactive oxygen species (ROS), creating an environment that can damage both native cells and transplanted stem cells. The oxidative stress can trigger apoptosis in stem cells, limiting their therapeutic potential. By delivering antioxidants or signaling molecules, ADSC exosomes could enhance the viability of transplanted stem cells in the ischemic brain. This could improve the overall outcomes of stem cell therapy in AIS.

### 8.8 circFUNDC1

Bai et al. (2022) investigated the role of circRNA FUN14 domain containing 1 (circFUNDC1) in human brain microvascular endothelial cells under oxygen-glucose deprivation conditions, mimicking ischemic stroke. CirculFUNDC1 expression was increased in IS patients and OGD-treated HBMECs, and knockdown alleviated OGD-induced cell apoptosis and enhanced cell viability, migration, and angiogenesis. CirculFUNDC1 also acted as a sponge for miR-375, with restoration having similar effects. The overexpression of PTEN counteracted this effect (Bai et al., 2022).

### 8.9 CircOGDH

CircOGDH, a circRNA generated from oxoglutarate dehydrogenase, was studied by Liu Y. et al. (2022) as a possible biomarker and treatment target for AIS. AIS is a severe neurological condition that can lead to disability and mortality. The main goal of treating AIS is to restore blood flow to the affected area within a specific time frame to save the penumbra, the reversible brain tissue surrounding the stroke core. CircOGDH, an upregulated gene in the penumbra tissue of mice with MCAO, has potential as a diagnostic biomarker for the penumbra in patients with AIS. CircOGDH interacts with miR-5112 in primary cortical neurons, enhancing COL4A4 expression, promoting neuronal damage and apoptosis under ischemic conditions. The findings suggest that targeting the CircOGDH may have a protective effect on neurons affected by ischemia. This is evidenced by the increased survival of neuronal cells observed when CircOGDH expression was knocked down (Liu Y. et al., 2022).

## 9 Glioblastoma

Glioblastoma (GBM) is a malignant type of brain cancer that originates from supportive cells in the brain called glial cells. Among adults, it is the most common and deadliest form of primary brain tumor. GBM presents obstacles to treatment, such as behavior, resistance to therapies, and the existence of the BBB, which restricts the delivery of drugs to tumor. NcRNAs derived from stem cells present possibilities for addressing these difficulties. They have the ability to focus on glioma cells and effectively penetrate the tumor core. Additionally, ncRNAs can modulate the functioning of genes associated with drug resistance, potentially making tumor cells more responsive to chemotherapy or other targeted treatments. Moreover, exosomes originating from stem cells have the ability to cross the BBB, enabling the delivery of ncRNAs to the location of the tumor. Scientists have discovered ncRNAs that have significant involvement in the development and advancement of GBM, and there are ongoing endeavors to leverage their potential as therapeutic agents. Gene editing techniques can be used to modify or alter stem cells to enhance their capabilities. One way to achieve this is by introducing coding RNAs or releasing exosomes containing these RNAs. By doing so, scientists can precisely deliver ncRNAs to GBM cells, which may help minimize unintended effects on other cells.

### 9.1 miR-124-3p

Zhang L. et al. (2024) demonstrated that NSC-EXOs can effectively transport miR-124-3p to GBM cells, inhibiting their proliferation, invasion, and migration. The exosomes can be modified to target GBM cells, allowing direct delivery of miR-124-3p to the tumor site. The researchers found that miR-124-3p targeted the flotillin 2 (FLOT2) gene in gliomas, suppressing FLOT2 expression and affecting the AKT1 pathway. The study also demonstrated that NSC-EXOs loaded with miR-124-3p effectively inhibited glioma growth by activating the EXO-miR-124-3p/FLOT2/AKT1 pathway. The authors of this study suggested the potential of NSC-EXOs as a delivery system for miR-124-3p to inhibit glioma growth, offering insights into stem cell-free molecular targeted therapy for glioma treatment (Zhang L. et al., 2024).

### 9.2 miR-133b

Xu et al. (2019) discovered that miR-133b, a miRNA derived from MSCs, exerts a regulatory influence on the expression of the cancer-associated gene EZH2. Glioma cell migration, invasion, and proliferation were all decreased by the inhibition of EZH2. The researchers also examined the impact of MSC-Exos harboring miR-133b on glioma cells. miR-133b-containing MSC-Exos blocked glioma cell invasion, migration, and proliferation by inhibiting EZH2 and the Wnt/β-catenin signaling pathway. These tumor-suppressive effects were confirmed by *in vivo* tests, which implies that exosomes may be used as biomarkers to treat gliomas (Xu et al., 2019).

### 9.3 miR-199a

Researchers have investigated the role of miR-199a in GBM, specifically when it is carried through exosomes derived from MSCs. Exosomes released from MSCs can transport miR-199a, a miRNA that can inhibit the growth and progression of glioma by targeting and regulating the expression of a gene called AGAP2. Yu et al. (2019) found that miR-199a had lower expression levels, while AGAP2 showed higher expression levels in glioma. The researchers identified AGAP2 as a potential target gene for regulation by miR-199a. To test their hypothesis, the researchers transfected MSCs with a miR-199a mimic, and then collected the exosomes that were derived from these genetically modified MSCs. They co-cultured these exosomes with glioma cells (U251 cells) and assessed their biological behaviors and chemosensitivity. The findings indicated that the exosomes successfully transported miR-199a to the glioma cells, reducing their growth, invasion, and migration. MSCs with increased expression of miR-199a improved the sensitivity of glioma cells to temozolomide. This study suggests that miR-199a could be a potential treatment for glioma (Yu et al., 2019).

### 9.4 miR-944

Exosomal miR-944 derived from glioma stem cells (GSCs) has been studied for its role in glioma progression and blood vessel formation. Individuals exhibiting lower levels of miR-944 experienced shorter overall survival periods, and high-grade gliomas exhibited lower miR-944 levels than low-grade gliomas. Jiang et al. (2021) *in vitro* research demonstrated that GSC-derived miR-944 suppresses HUVECs migration, proliferation, and tube formation. In mouse studies, the researchers found that agomiR-944 (a miR-944 agonist) significantly reduced tumor growth and angiogenesis. Exosomes derived from GSCs carry a miRNA called miR-944, which reduces VEGFC expression and inhibits the AKT/ERK signaling pathway in HUVECs and in xenograft glioma cell tumors (Jiang et al., 2021).

## 10 Brain injury

Brain injury occurs when the brain is injured, resulting in impaired functioning. Traumatic brain injury (TBI) and acquired brain injury (ABI) are the two primary categories of brain injuries. TBI occurs when the brain is injured due to an impact on the head or a penetrating injury, which disrupts brain function. On the other hand, ABI refers to brain damage occurring after birth due to traumatic causes, such as stroke, infection, oxygen deprivation, or tumors. The impact and outcomes of brain injuries can differ significantly, ranging from minor to major. Minor brain injuries, such as concussions, might cause symptoms such as headaches, dizziness, confusion, or memory issues. In contrast, when someone experiences a moderate to severe brain injury, it can result in long-lasting or permanent difficulties in physical, cognitive, emotional, and behavioral functioning. These difficulties may appear as problems with movement and coordination, speaking, remembering things, staying focused, concentrating, solving problems, effectively controlling emotions, or maintaining a mood. They can also cause changes in personality or mood.

### 10.1 miR-124

Huang W. et al. (2023) investigated the possibility of using bone marrow MSC-Exos to treat ischemic stroke. They found that MSC-Exos can enhance the expression of glutamate transporter 1 (GLT-1) in response to ischemic stroke. The researchers used MSC-Exos, miR-124 inhibitors and mimics, and rapamycin (mTOR pathway inhibitor) to investigate the mechanism. Huang et al. (2021) showed that MSC-Exos prevented the decrease in GLT-1 and miR-124 expression and increased pS6 expression in astrocytes after oxygen-glucose deprivation/reperfusion. This study suggests that MSC-Exos have therapeutic potential against ischemic stroke by regulating GLT-1 expression, with miR-124 and the mTOR pathway playing a role (Huang W. et al., 2023).

### 10.2 miR-150-5p

In a rat model of MCAO, researchers explored how pathogenic changes, neuronal apoptosis, inflammatory indicators, and neurological function were affected by exosomal miR-150-5p derived from bone marrow MSCs. In Li et al.'s research (Li et al., 2022), BMSC-Exos was isolated, recognized, and administered these exosomes to MCAO rats. They demonstrated that miR-150-5p expression declined in MCAO rats and TLR5 levels increased. However, BMSCs-Exos treatment enhanced neurological function, diminished pathological alterations, reduced neuron apoptosis, and lessened inflammatory factors. Neurological function was enhanced, pathological alterations were diminished, neuron apoptosis was lowered, and inflammatory factors were lessened with BMSC-Exos treatment. The protective effects of BMSCs-Exos against cerebral ischemia/reperfusion (I/R) damage were improved by the enrichment of miR-150-5p (Li et al., 2022).

### 10.3 miR-223-3p

Researchers have found that exosomal miR-223-3p derived from MSCs may help decrease brain ischemia and inflammation caused by the M1 polarization of microglia. Zhao et al. (2020) conducted a study using BV-2 microglia stimulated with cysteinyl leukotrienes (CysLTs) and rats that had undergone MCAO/R surgery. In rats with MCAO/R-induced cerebral ischemia, exosomes produced from MSCs transfected with miR-223-3p alleviated neurological impairment, decreased cerebral infarct volume, and improved learning and memory capacities. Exosomes also reduced the expression of CysLT2R, a type of brain cell with anti-inflammatory properties. This phenomenon suggests that exosomal miR-223-3p originating from MSCs helps to lessen the impact of I/R injury. miR-223-3p does this by inhibiting the polarization of cells toward the M1 phenotype and suppressing the overall inflammatory reaction. This beneficial effect may be linked to the way miR-223-3p inhibits the CysLT2R receptor (Zhao et al., 2020).

### 10.4 miR-150-3p

Researchers have studied the protective role of exosomes containing miR-150-3p from NSCs concerning hypoxia and ischemia-induced brain injury. The study by Luo et al. (2022) demonstrated that under both standard conditions and oxygen-glucose deprivation conditions, NSCs-Exo increased cell proliferation and prevented apoptosis in SH-SY5Y cells. Using a middle cerebral artery occlusion model, *in vivo* experiments revealed that NSCs-Exo inhibited neuronal death and decreased infarction area. It was discovered that miR-150-3p, the most prevalent in exosome miRNA, has neuroprotective properties. According to the study, miR-150-3p targets CASP2 and suppresses apoptosis following brain damage, emphasizing the potential of NSCs-Exo as a therapeutic approach for cerebral injury (Luo et al., 2022).

### 10.5 miR-124-3p

Exosomes produced by BMSCs have been discovered by researchers to have an impact on inflammation, synaptic plasticity, and neuronal proliferation in the brain, which may affect the pathophysiology of TBI. Zhuang et al. (2023) found that rats with TBI exhibited decreased miR-124-3p levels but increased p38 MAPK levels. Exosomes derived from miR-124-3p-treated BMSCs reduced glutamate-mediated excitotoxicity by upregulating GLT-1 expression and downregulating p38 MAPK. This therapy increased neurological function, decreased lesion volume, and decreased the death of neurons. These results highlight the potential of exosomal miR-124-3p as a therapeutic strategy for treating TBI (Zhuang et al., 2023).

### 10.6 miR-17–92

Zhang Y. et al. (2021) investigated how exosomes carrying miR-17-92 affected TBI. The researchers compared the efficacy of exosomes generated from MSCs that had been transfected with a control vector, versus exosomes that were enhanced to overexpress the miR-17–92 cluster. The study involved adult male rats who underwent unilateral moderate cortical contusion to simulate TBI. Rats were divided into three groups: Exo-17-92, Exo-empty, and control, and their neurological function was assessed 5 weeks after TBI. Both interventions boosted angiogenesis and neurogenesis, decreased neuroinflammation, and markedly enhanced sensorimotor and cognitive performance. The study found that the exosomes overexpressing the Exo-17-92 exerted a more substantial therapeutic effect compared to the control exosomes (Exo-empty). Specifically, the Exo-17-92 exosomes enhanced functional recovery, reduced neuroinflammation, and reduced cell loss to a greater degree. Exosomes enriched with the miR-17–92 cluster reduced neuroinflammation and enhance endogenous angiogenesis and neurogenesis, thereby improving functional recovery following TBI (Zhang Y. et al., 2021).

### 10.7 miR-132-3p

miR-132-3p-primed exosomes made from MSCs have been shown by Pan et al. (2020) to enhance the prognosis of patients with brain ischemia damage. The exosomes, which were primed with miR-132-3p, a miRNA involved in neuronal survival and plasticity, had a more significant impact on promoting neuronal cell survival and proliferation. They also showed increased expression of neuroprotective and angiogenic factors, which are crucial for neuronal health and blood vessel formation. *In vivo* experiments showed reduced brain damage, improved neurological function, and increased blood vessel formation in mice treated with primed exosomes. However, further research is needed to understand their effects and assess their long-term safety and effectiveness (Pan et al., 2020).

### 10.8 lncRNA MALAT1

Exosomes generated from hASCs have been studied for their potential to treat TBI and aid recovery. Patel et al. (2018) found that exosomes containing MALAT1, a specific RNA, improved motor behavior and reduced brain injury in rats treated with hASCs. The results also revealed that MALAT1 in exosomes influences inflammation, cell cycle, cell death, and regeneration pathways (Patel et al., 2018). El Bassit et al. (2017) demonstrated in a different investigation that MALAT1 is essential for controlling neuronal survival and PKCδII splicing in HT22 cells. Exosomes derived from hASCs increased PKCδII expression, enhancing survival and proliferation. MALAT1 promoted alternative splicing and increased neuronal survival. Insulin treatment enhanced the association between MALAT1 and SRSF2 and improved HT22 cell survival and proliferation after injury. Combining exosome treatment with insulin therapy could enhance the regenerative effects in neuronal injury scenarios (El Bassit et al., 2017).

### 10.9 lncRNA KLF3-AS1

Xie et al. (2023) found that BMSC-Exos KLF3-AS1 can reduce CI and improve neurological function in mice and cell models exposed to OGD, mimicking cerebral I/R injury. The enzyme Sirt1 is blocked by KLF3-AS1 by inducing the synthesis of USP22, a protein that removes molecules from other proteins. KLF3-AS1 also acts as a sponge for miR-206, preventing its binding to USP22 mRNA and increasing its expression. This upregulation stabilizes the Sirt1 contributing to the protective effects of BMSC-Exos KLF3-AS1 (Xie et al., 2023).

## 11 Rett syndrome

Rett syndrome is a genetic disorder primarily affecting females that is characterized by severe cognitive and developmental impairments. Studies have been conducted to investigate the potential use of exosomes derived from stem cells and their cargo of ncRNAs in the context of Rett syndrome. Exosomes containing ncRNAs have been shown to affect spine growth, synaptic plasticity, and cognitive function in models of Rett syndrome. Pan et al. (2021) found that by controlling the EPha4/TEK axis, exosomal miR-21-5p generated from urine-derived stem cells (USC-Exos) may stimulate neurogenesis and mitigate Rett syndrome. They discovered that Eph receptor A4 gene, connected to neurogenesis, was the target of exosomal miR-21-5p in USC-Exos. The following treatment with USC-Exos, the proportion of nerve cells unique to individual neurons increased, and the expression of specific neural markers increased. In an *in vivo* model, the researchers observed improvements in behavior, motor coordination, and cognitive abilities in treated mice. This study provides evidence that exosomal miR-21-5p can also help in neural cells differentiation (Pan et al., 2021).

## 12 Epilepsy

Epilepsy is a condition that affects the body that leads to repeated seizures. Researchers are actively investigating the use of stem cell-derived exosomes, which contain various ncRNAs, as a possible therapeutic method for treating epilepsy. Exosomes containing circHivep2 exert anticonvulsant effects, reduce seizure frequency, and promote neuronal survival and synaptic plasticity in epilepsy models. Scientists have discovered that exosomes containing circHivep2 derived from hASCs have potential as a therapeutic intervention for epilepsy. Xiaoying et al. (2020) focused on a specific circRNA called circHivep2 and its interaction with miR-181a-5p and SOCS2. Microarray analysis revealed that in mice with epileptic seizures induced by kainic acid, the expression of this gene was reduced in the hippocampus. Additionally, the authors discovered that circHivep2 controls SOCS2 expression by interacting with miR-181a-5p. *In vitro* studies have shown that circHivep2 overexpression suppressed inflammatory factors and microglial activation, whereas circHivep2 knockdown increased pro-inflammatory protein production and microglial activation (Xiaoying et al., 2020).

## 13 Diabetic peripheral neuropathy

Diabetes-related peripheral neuropathy (DPN) is a condition in which nerves in the limbs and organs are damaged due to diabetes. This condition is mainly caused by blood sugar levels, which damage nerve supply vessels and impair their function. Fan et al. (2021) explored using modified exosomes, specifically miR-146a, to treat DPN. Exo-146a, derived from cells, was created by altering exosomes derived from cells to contain miR-146a. Experiments in diabetic mice revealed that exo-146a had a high loading capacity for miR-146a and effectively accumulated in peripheral nerve tissues. The study found that treatment with exo-146a for a duration of 2 weeks led to significant improvements in nerve conduction velocity as well as reduced thresholds for thermal and mechanical stimuli. Additionally, it decreased inflammation by preventing blood endothelial cells and monocytes from becoming activated. The results suggest that engineered exosomes carrying miR-146a could be a promising therapeutic approach for DPN (Fan et al., 2021).

## 14 Conclusion

The use of ncRNAs as a therapeutic approach is a relatively new field that is still in the early stages of development and research. Initial research has displayed encouraging findings in studies suggesting that ncRNAs derived from stem cells could potentially affect neurological disease processes. Animal models of disorders have demonstrated the capacity of these ncRNAs to boost survival rates, stimulate neurogenesis, and enhance functional outcomes. However, further research and clinical studies are required to completely comprehend the potential, safety, and efficacy of ncRNAs-based therapies with exosomes produced from stem cells. However, there is a great promise for the creation of ground-breaking treatments for neurological illnesses through the use of ncRNAs extracted from exosomes produced by stem cells. These therapies can potentially provide substantial benefits to patients in the current era.
